# Multimeric structure of a subfamily III haloalkane dehalogenase‐like enzyme solved by combination of cryo‐EM and x‐ray crystallography

**DOI:** 10.1002/pro.4751

**Published:** 2023-10-01

**Authors:** Klaudia Chmelova, Tadeja Gao, Martin Polak, Andrea Schenkmayerova, Tristan I. Croll, Tanvir R. Shaikh, Jana Skarupova, Radka Chaloupkova, Kay Diederichs, Randy J. Read, Jiri Damborsky, Jiri Novacek, Martin Marek

**Affiliations:** ^1^ Loschmidt Laboratories, Department of Experimental Biology and RECETOX, Faculty of Science Masaryk University Brno Czech Republic; ^2^ International Clinical Research Center St. Anne's University Hospital Brno Brno Czech Republic; ^3^ Central European Institute of Technology Masaryk University Brno Czech Republic; ^4^ Department of Hematology, Cambridge Institute for Medical Research University of Cambridge Cambridge UK; ^5^ Institute of Neuropathology University Medical Center Göttingen Göttingen Germany; ^6^ Department of Biology University of Konstanz Konstanz Germany

**Keywords:** catalysis, cryo‐EM, DhmeA, haloalkane dehalogenase, *Haloferax mediterranei*, multimerization, x‐ray crystallography

## Abstract

Haloalkane dehalogenase (HLD) enzymes employ an S_N_2 nucleophilic substitution mechanism to erase halogen substituents in diverse organohalogen compounds. Subfamily I and II HLDs are well‐characterized enzymes, but the mode and purpose of multimerization of subfamily III HLDs are unknown. Here we probe the structural organization of DhmeA, a subfamily III HLD‐like enzyme from the archaeon *Haloferax mediterranei*, by combining cryo‐electron microscopy (cryo‐EM) and x‐ray crystallography. We show that full‐length wild‐type DhmeA forms diverse quaternary structures, ranging from small oligomers to large supramolecular ring‐like assemblies of various sizes and symmetries. We optimized sample preparation steps, enabling three‐dimensional reconstructions of an oligomeric species by single‐particle cryo‐EM. Moreover, we engineered a crystallizable mutant (DhmeA^ΔGG^) that provided diffraction‐quality crystals. The 3.3 Å crystal structure reveals that DhmeA^ΔGG^ forms a ring‐like 20‐mer structure with outer and inner diameter of ~200 and ~80 Å, respectively. An enzyme homodimer represents a basic repeating building unit of the crystallographic ring. Three assembly interfaces (dimerization, tetramerization, and multimerization) were identified to form the supramolecular ring that displays a negatively charged exterior, while its interior part harboring catalytic sites is positively charged. Localization and exposure of catalytic machineries suggest a possible processing of large negatively charged macromolecular substrates.

## 1 INTRODUCTION

Haloalkane dehalogenases (EC 3.8.1.5, HLDs) are a group of enzymes that catalyze the hydrolytic cleavage of a carbon–halogen bond in a range of halogenated organic compounds. The HLD family is thoroughly studied with a wide range of biotechnological applications including industrial biocatalysis, toxic by‐product recycling, decontamination of chemical warfare agents, biosensing and bioremediation of environmental pollutants, and protein tagging for cell imaging (Koudelakova et al., 2013). They can be found in all domains of life; prokaryotic archaea and bacteria as well as eukaryotic organisms (Chovancova et al., 2007). However, their fundamental biological role in cellular processes remains unclear to this day.

Structurally, HLDs are categorized under the α/β‐hydrolase superfamily, a diverse group of hydrolytic enzymes (Dimitriou et al., 2017). The canonical αβα‐sandwich is an integral part of the haloalkane dehalogenase architecture. Another vital component of the HLD molecule is the helical cap domain, the structure of which has an impact on the substrate specificity of many α/β‐hydrolase fold enzymes (Dimitriou et al., 2017). A distinct catalytic pentad forms the active site of HLD enzymes: a nucleophile aspartic acid, a histidine base, a catalytic acid (aspartic or glutamic acid) and two halide stabilizing residues (two tryptophans or tryptophan + asparagine) (Janssen, 2004). The active site of HLDs is situated in a hydrophobic pocket between the α/β‐fold core and the cap domain, connected to the surrounding solvent via a main tunnel and a slot tunnel (Markova et al., 2020; Pavlova et al., 2009).

The most thoroughly described HLD function is the conversion of alkyl halides to their corresponding alcohols, halides, and a proton. This two‐step catalytic process consists of an S_N_2 nucleophilic substitution followed by hydrolysis of the ester intermediate (Verschueren et al., 1993). Water is the only required co‐factor for this reaction (Damborsky & Koča, 1999). The substrate array of HLDs encompasses a large variety of halogenated aliphatic hydrocarbons, such as alkanes, cycloalkanes, alkenes, ethers, alcohols, ketones, and cyclic dienes (Damborsky et al., 2001). The HLD family has therefore been divided into four substrate specificity groups (SSG). The preference for certain substrates seems to be influenced by the architecture of the access tunnels and the active site, rather than the sequence similarity across the HLD family (Koudelakova et al., 2013; Pavlova et al., 2009). This categorization of HLDs is vital for further bioengineering efforts. Nevertheless, many of the tested organohalogen substrates were synthetic, and therefore the SSG classification does not necessarily reflect the *in vivo* function of these enzymes.

Another approach to classify HLDs utilizes an evolutionary perspective. Phylogenetic analyses of the haloalkane dehalogenase family revealed three distinct subfamilies: HLD‐I, HLD‐II, and HLD‐III. These three subfamilies vary mainly by the composition of their catalytic pentad and the structure of their cap domain (Chovancova et al., 2007). The first two subfamilies contain the three archetypal HLDs: DhlA from HLD‐I, and DhaA and LinB from HLD‐II. These three renowned enzymes have been identified decades ago and paved the way for the engineering and biotechnology utilization of HLDs (Nagata et al., 2015). Numerous crystal structures of the members of the HLD‐I and HLD‐II families have been determined, partially due to their ease of handling and overall stability (Chaloupkova et al., 2014; Franken et al., 1991; Gehret et al., 2012; Lahoda et al., 2014; Marek et al., 2000; Mazumdar et al., 2008; Oakley et al., 2004; Verschueren et al., 1993).

Comparatively, subfamily III (HLD‐III) is the least studied of the three HLD subfamilies. Numerous members originate from pathogenic or extremophilic organisms and display low dehalogenase activity compared to the other two subfamilies (Vanacek et al., 2018; Vasina et al., 2022). Therefore, haloalkane dehalogenase‐like enzymes is a more accurate term for the members of the HLD subfamily III. Prominent examples that have been previously characterized include DrbA from *Rhodopirellula baltica*, DmbC from *Mycobacterium bovis*, and DmrB extracted from *Mycobacterium rhodesiae* (Fung et al., 2015; Jesenska et al., 2009). While maintaining low dehalogenase activity, there are promiscuous members of the HLD‐III subfamily that have been identified. A notable example of such a putative HLD‐III is OleB, an enzyme from *Xanthomonas campestris* that acts as a β‐lactone decarboxylase in the biosynthetic pathway of long‐chain olefinic hydrocarbons (Christenson, Jensen, et al., 2017).

Attempts in experimental characterization revealed many practical limitations to working with the HLD‐III subfamily members, namely low‐expression yields, low solubility of the recombinant proteins, multimerization, and high polydispersity of the protein samples (Fung et al., 2015; Jesenska et al., 2009; Koudelakova et al., 2013; Vanacek et al., 2018). These circumstances undeniably contribute to the main challenge of studying the HLD‐III enzymes; no high‐resolution structure has yet been published for this subfamily of enzymes.

DhmeA is yet another member of the elusive HLD‐III subfamily (Chovancova et al., 2007). DhmeA originates from the extremophilic archaeon *Haloferax mediterranei* that inhabits hypersaline water ecosystems, such as saltwater evaporation ponds (Han et al., 2012; Oren & Hallsworth, 2014). Previous experimental characterizations confirmed many of the commonly observed features of the HLD‐III family: low‐expression yield and poor solubility, oligomerization tendency and polydisperse heterogeneity of the final product, as well as low dehalogenase activity (Vanacek et al., 2018; Vasina et al., 2022). One of its unique features is the melting temperature of 71°C, currently making it the most natively thermostable wild‐type HLD (Vanacek et al., 2018).

In this work, we report insight into the structural organization of the DhmeA protein. Collectively, we present an integrative structural biology approach, combining cryo‐EM and x‐ray crystallography, to obtain the structural snapshots and multimerization potential of an HLD‐III enzyme. This was accomplished via optimization of the sample preparation process while considering its halophilic origin. Numerous oligomeric and multimeric states of wild‐type DhmeA were visualized by cryo‐electron microscopy (cryo‐EM), where the protein dimers were repeating units in the building of multimeric assemblies. A similar mode of multimerization was also observed in a 3.3 Å crystal structure of an engineered DhmeA variant (DhmeA^ΔGG^), which adopts a ring‐like 20‐mer structure. The ring exhibits a highly negatively charged exterior and positively charged interior, where catalytic machinery is located. Additionally, every second monomeric unit of the ring has an exposed catalytic machinery by a partially unfolded cap domain which is responsible for a substrate specificity and substrate entrance. DhmeA^ΔGG^ ring anatomy and charge distribution show a certain resemblance to enzymes binding DNA and/or nucleotides (recombinases). Therefore, we anticipate that the biological function of DhmeA might be the catalysis of bulky, negatively charged substrates. Although there is little known about halogenation/dehalogenation mechanisms in bacteria/archaea, halogenated nucleotides were found even in non‐halophilic cells, for example, human cells (Henderson et al., 2001, 2003; Valinluck et al., 2005). Therefore, there might be a need in many organisms for enzymes specialized in dehalogenation of nucleotides/DNA.

## 2 RESULTS

### 2.1 Wild‐type DhmeA is a multimeric protein with a tendency to form ring‐like structures

**FIGURE 1:**
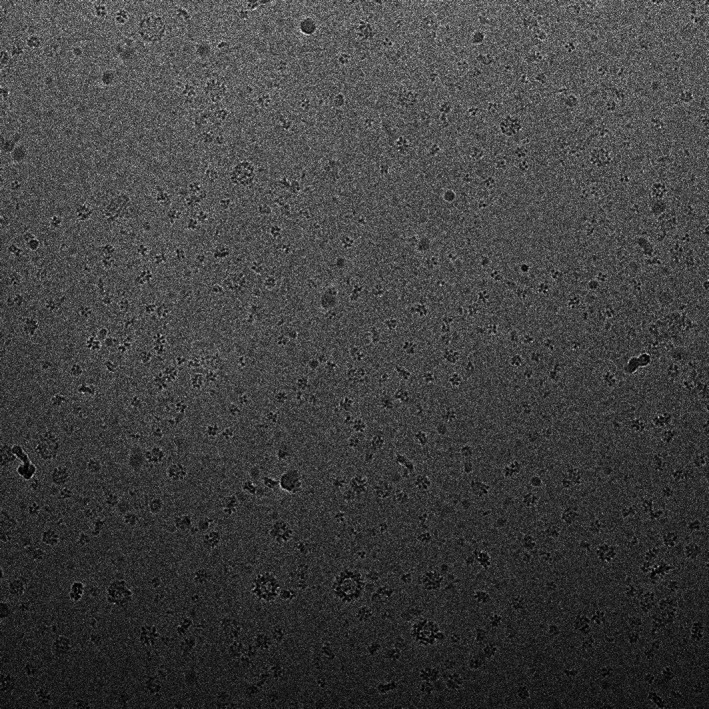
Cryo‐EM micrograph of full‐length wild‐type DhmeA, showing structural heterogeneity and high multimerization potential of the protein (magnification: 50,000×).

We used a single‐particle cryo‐EM technique to probe the multimerization potential of wild‐type DhmeA. As shown in Figure 1, the sample of recombinantly produced and affinity‐purified wild‐type DhmeA is structurally highly heterogeneous, containing oligomeric and multimeric assemblies of various sizes. We thus picked each particle semi‐manually and created a dataset containing 6,294 particles in multiple orientations or conformations. After that, similar images were computationally aligned to each other and averaged in order to obtain the images with a better signal‐to‐noise ratio. Finally, classification was performed to group similar particles into different classes, resulting in 29 different classes of 2D projections of wild‐type DhmeA quaternary structure assemblies (Table 1). Among them, we could observe DhmeA homo‐multimers of various symmetries, ranging from trimers, tetramers, pentamers, hexamers, heptamers, and octamers to high‐ordered ring‐like supramolecular structures. Collectively, the preliminary cryo‐EM imaging showed that the wild‐type DhmeA is a multimerization‐prone protein that forms diverse and polydisperse macromolecular assemblies, preventing its structural characterization by high‐resolution techniques such as x‐ray crystallography and single‐particle cryo‐EM.

**TABLE 1:** Identified classes of wild‐type DhmeA quaternary structure assemblies and their populations.

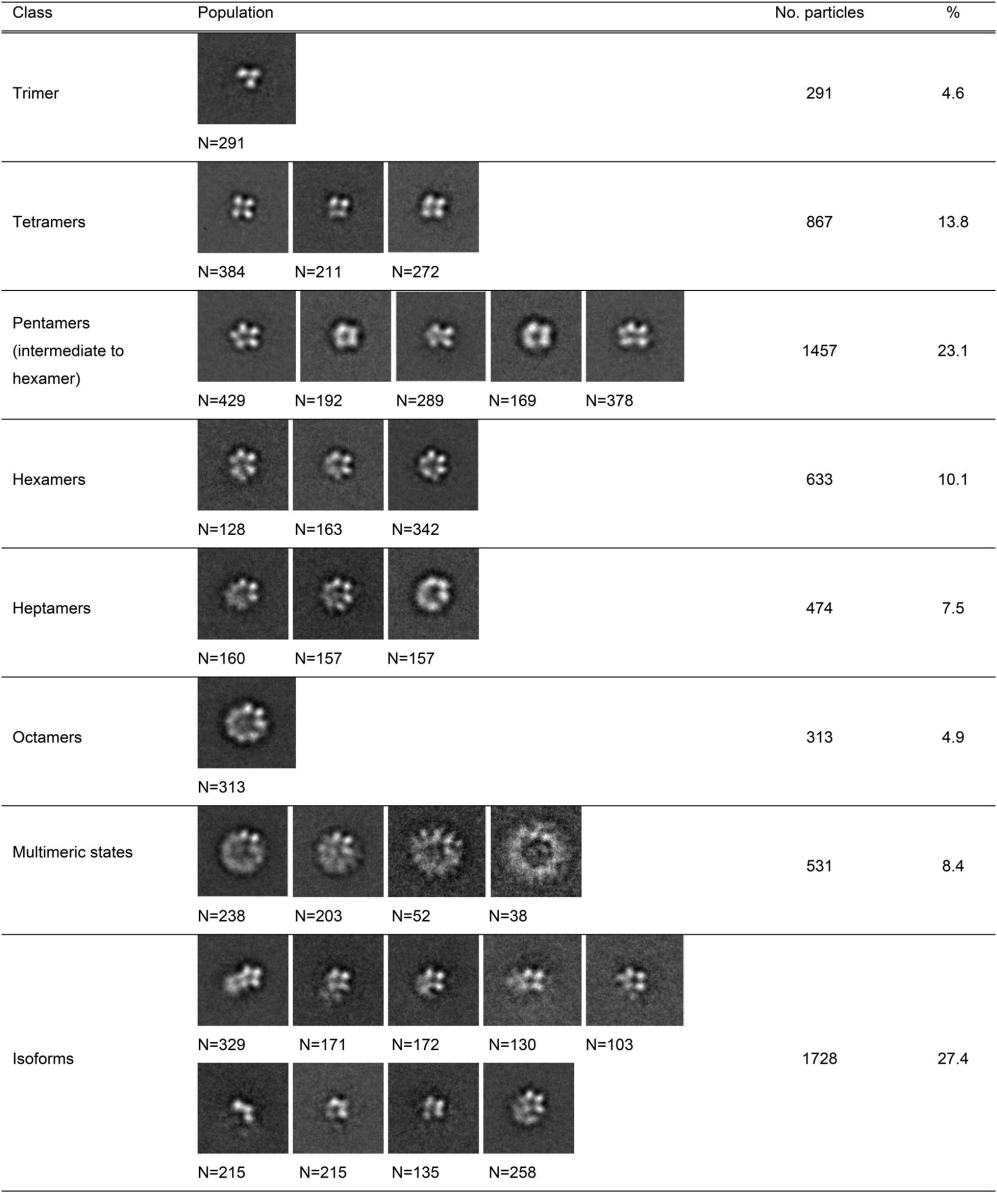

### 2.2 Optimized purification of wild‐type DhmeA

**FIGURE 2:**
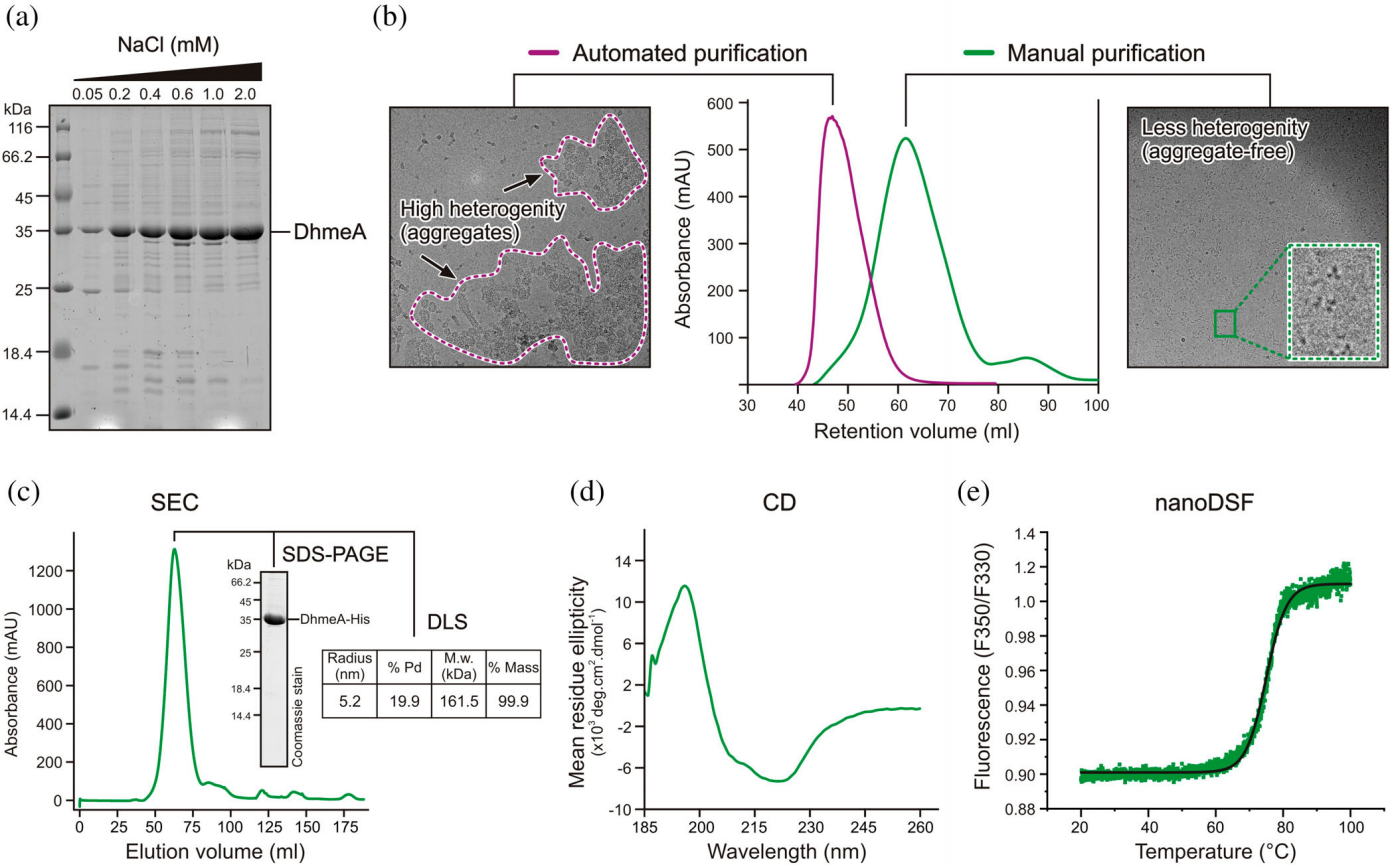
Optimized purification and characterization of wild type DhmeA. (a) Mini‐scale expression and purification of DhmeA using buffers with increasing salt concentration (from 0.05 to 2 M NaCl). SDS‐PAGE analysis shows that the solubility of DhmeA correlates with increased salt concentration. (b) Automated FPLC‐based versus manual gravity flow affinity chromatography effects on quality of DhmeA sample. Size‐exclusion chromatography (Sephadex S200 gel filtration column) experiments show a difference in sample sizes. EM analyses of corresponding fractions show that the automated FPLC‐based affinity chromatography yields highly heterogenous DhmeA complexes of various sizes and shapes, which is not the case for manual, gravity flow chromatography purification. (c,d) Quality control of DhmeA purified through the optimized manual purification and assayed by SDS‐PAGE and DLS measurement (c), CD spectroscopy (d) and differential scanning fluorimetry (e).

We noted that a high yield and solubility of DhmeA positively correlate with increasing ionic strength of the purification buffer. Specifically, we demonstrated that the highest solubility of DhmeA was achieved in the buffer containing 2 M NaCl (Figure 2a), which may reflect its extremophilic origin. However, 2 M NaCl is not only too high for the efficient use of metal affinity chromatography, but it could as well interfere with data collection in cryo‐EM. Therefore, in our next experiments, we used the purification buffer with 1 M NaCl as a compromise.

DhmeA was purified as high‐molecular‐weight and often aggregated multimers when eluted from a Superdex S200 gel filtration column following automated FPLC‐based immobilized metal affinity chromatography (IMAC). EM imaging revealed that these complexes of wild‐type DhmeA have various sizes and shapes, indicating that the sample was still too heterogenous for further structural analysis (Figure 2b). We found out that the use of automated FPLC‐based IMAC contributes to the formation of these highly heterogenous and polydisperse DhmeA complexes due to high pressure. For this reason, we switched to a manual, bench‐based gravity flow IMAC setup that provides lower pressure and therefore fewer heterogenous DhmeA complexes of lower molecular weights, better suited for structural characterization (Figure 2b). Biophysical characterization of the wild‐type DhmeA complexes purified by this optimized protocol showed a good polydispersity (<20%), proper folding and high thermal stability (*T*_m_ = 76°C) (Figure 2c–e). DLS experiments estimated an average particle radius of wild‐type DhmeA complexes of ~5.2 nm, with an approximate molecular weight (MW) of ~161.5 kDa. With the theoretical MW of the monomeric DhmeA, ~35.8 kDa, the DLS measurements suggest that DhmeA could form homotetramers (theoretical MW for DhmeA tetramer is ~143 kDa). Here, it is important to highlight that the elution peak upon the gel filtration (Superdex S200 column) is rather broad, indicating the presence of multiple oligomeric species.

The correct folding and the secondary structure of wild‐type DhmeA were verified by CD spectroscopy in the far‐UV spectral region. The enzyme exhibited similar CD spectra to other previously characterized HLDs harboring the α/β‐hydrolase fold (Babkova et al., 2017; Franken et al., 1991; Ollis et al., 1992; Shan et al., 2021). Together, the optimized purification procedure provides wild‐type DhmeA enzyme in quantity and quality suitable for structural studies.

### 2.3 Cryo‐EM structure of the wild‐type DhmeA complexes

**FIGURE 3:**
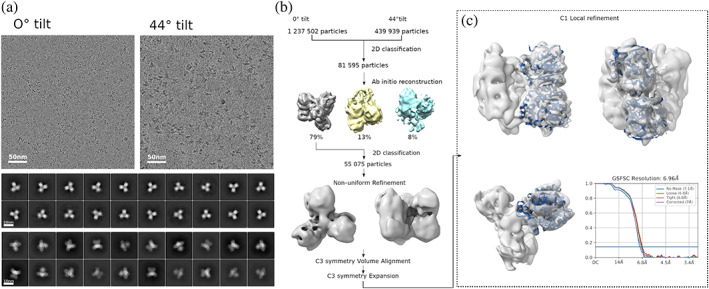
Cryo‐EM structure of the full‐length DhmeA. Cryo‐EM data were collected at 0° and 44° stage tilt with 1,237,502 and 439,939 particles extracted, respectively (a). After the 2D classification, 81,595 particles were selected for cryoSPARC *ab initio* reconstruction with three models. The model with 64,146 particles was processed with another 2D classification to balance the distribution of differently oriented particles continued with C1 Non‐uniform Refinement. The dataset was subsequently aligned and expanded with C3 symmetry to effectively increase the dataset size (b). The local refinement resulted in the final cryo‐EM density of the DhmeA dimer at 7 Å (EMDB ID: EMD‐17015) (c).

The cryo‐EM data collected on optimized DhmeA sample showed particles with tentative threefold symmetry (Figure 3). The initial data analysis revealed significant orientational preference of the particles in the ice. Therefore, we have used a strategy addressing the issue of non‐uniform particle orientation distribution by combining data collected at different sample tilt (Tan et al., 2017). The cryo‐EM map reconstructed from the major class of the particles present in the data showed six DhmeA molecules arranged into a D3 assembly. The resolution of the final model was limited to 7–8 Å which limited further data analysis. We have tested both focused refinement and focused classification of a single DhmeA cryo‐EM density. None of the focused data refinement strategies led to improvement of model resolution which suggests that wild‐type DhmeA forms a highly dynamic assembly.

No individual secondary structure elements were clearly defined in the cryo‐EM map, with the exception of a central eight‐stranded β‐sheet that could be unambiguously located. The structure is built from three DhmeA dimers, which are arranged around a 3‐fold symmetry axis. The dimerization interface seems to be tightly packed, while inter‐dimer contacts are less tight. We therefore deduce that the dimeric unit is a common building block of large assemblies formed by wild‐type DhmeA.

### 2.4 Engineering the DhmeA^ΔGG^ to favor its crystallization

Since cryo‐EM did not yield a high‐resolution structure, we initiated crystallization experiments with DhmeA. Attempts to crystallize the full‐length wild‐type DhmeA yielded only irreproducible, and poorly diffracting crystals. We thought that this was mainly due to (i) the high heterogeneity of the sample as observed in cryo‐EM and (ii) the high conformational flexibility of the DhmeA protein. In our previous work we learned that a helical cap domain is the most flexible and malleable part of the HLD‐fold proteins (Markova et al., 2021; Schenkmayerova et al., 2023). We noted that the solvent‐exposed L10 loop, connecting α4 and α5' helices in the cap domain, displays a unique feature containing two glycine residues (G173, G174) in DhmeA. We hypothesized that this glycine‐rich loop could be responsible for the high conformational flexibility of the wild‐type DhmeA. We thus constructed a DhmeA mutant lacking these two glycines, hereafter referred to as DhmeA^∆GG^. The DhmeA^∆GG^ turned out to be more soluble when recombinantly expressed in *E*. *coli*, and more importantly, it was purified in higher homogeneity than its wild‐type counterpart. Importantly, the DhmeA^∆GG^ protein could be reproducibly crystallized. Although diffraction was generally weak, x‐ray data were collected, and several full data sets reached up to 3.3 Å resolution.

### 2.5 Crystal structure of DhmeA^ΔGG^ reveals a 20‐mer ring‐like assembly

The structure was solved by a multi‐step molecular replacement procedure (see Methods for details), using a modeled DhmeA^∆GG^ dimer. Due to the large unit cell (*a* = 172.76 Å, *b* = 289.9 Å, *c* = 168.31 Å; α = β = γ = 90°) and the low resolution of crystallographic data (~3.3 Å), the molecular replacement process was challenging, but the availability of the cryo‐EM map made this possible. In the first step, we generated a homology model of DhmeA^∆GG^ that was then docked into the low‐resolution cryo‐EM map, and further optimized with ISOLDE (Croll, 2018) help, to retrieve a homodimeric unit. With the correctly positioned homodimeric model structure, we could then locate all five homodimers in the asymmetric unit through rotational and translation searches. The initial model was further refined by several cycles of manual building and automated refinement. The final model contains 10 DhmeA molecules in the asymmetric unit and has a low deviation from the ideal geometry (Table S1).

**FIGURE 4:**
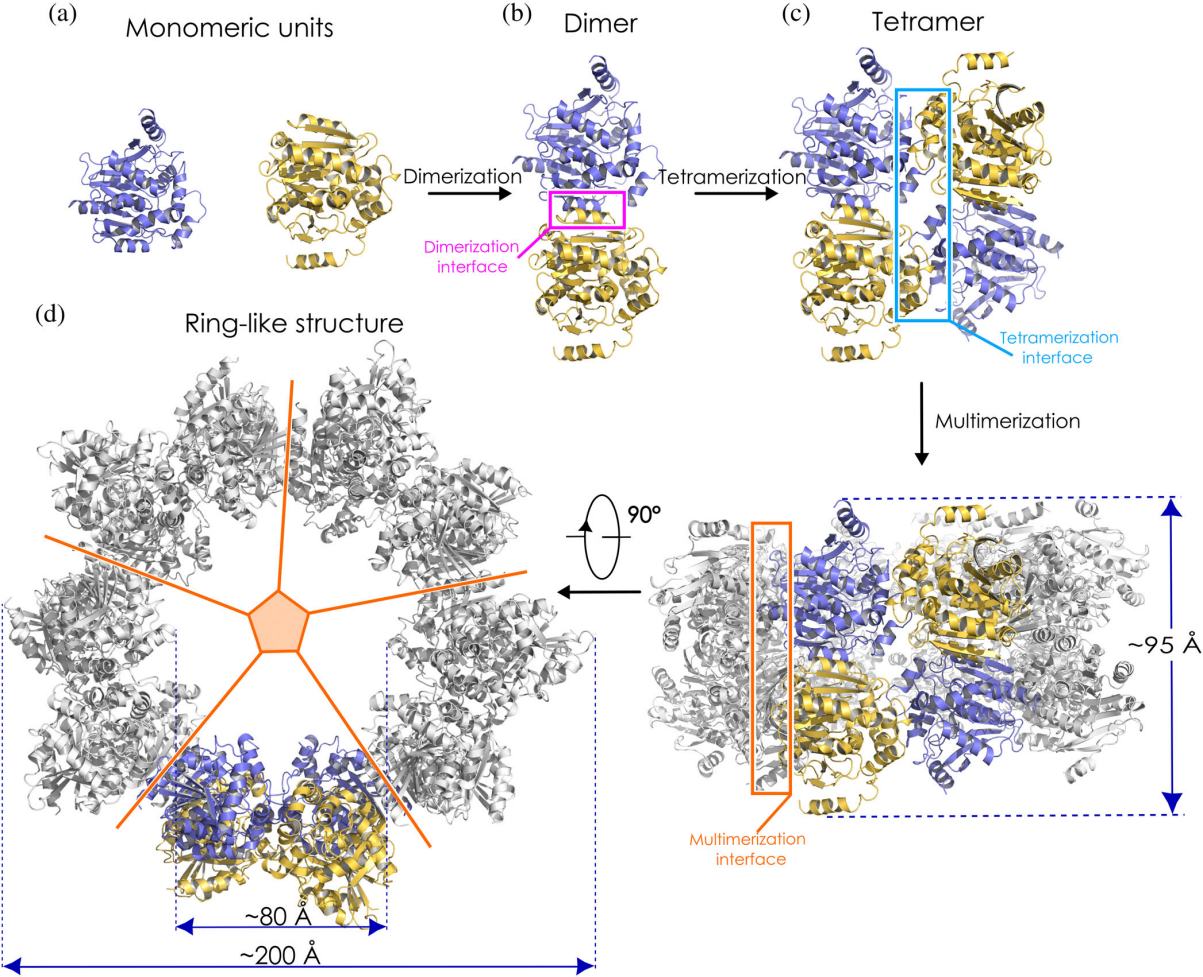
Cartoon representation of the overall crystal structure of DhmeA^ΔGG^. Two monomers (a) form a homodimer (b). Two homodimers associate into a homotetramer (c). Five homotetramers are arranged around fivefold symmetry axis, assembling crystallographic ring‐like 20‐mer structure (d). The inner diameter of the ring measures ~80 Å, outer diameter ~200 Å, while the height of the ring is ~95 Å. The monomeric units are colored as yellow and blue. PDB ID accession code: 8CKP.

The crystal structure of the DhmeA^ΔGG^ reveals molecular details of its multimerization potential (Figure 4). Two monomeric units associate with each other, forming a homodimeric unit. Ten homodimers assemble into a ring‐like structure. The resulting ring‐like structure is built up from 20 DhmeA^ΔGG^ molecules. The 20‐mer ring displays five‐fold symmetry, with an inner diameter of the ring of ~80 Å, an outer diameter of ~200 Å, and a ring height is ~95 Å (Figure 4). The crystallographic ring contains three types of assembly interfaces, namely di‐, tetra‐, and multimerization interface. The dimerization interface connects two monomeric units into a homodimer. The crystallographic ring represents five‐fold symmetry (Figure 4d), meaning that two homodimers can be presented as a homotetramer. The interface between two homodimers is referred to as a tetramerization interface. Five homotetramers finally form a crystallographic ring‐like structure via a so‐called multimerization interface. One should note that the above‐mentioned order of assembling (dimer → tetramer → ring‐like structure) does not necessarily represent the actual order of assembling *in vivo*. The supramolecular ring‐like arrangement found in the lattice of DhmeA^ΔGG^ crystals resembles some multimeric ring‐like structures observed by cryo‐EM imaging with the full‐length wild‐type DhmeA (Table 1). We therefore think that the structural features observed in the x‐ray structure of DhmeA^ΔGG^ reflect to some extent a behavior of the wild‐type protein.

### 2.6 Zooming in on monomeric units: Buried versus exposed active site

**FIGURE 5:**
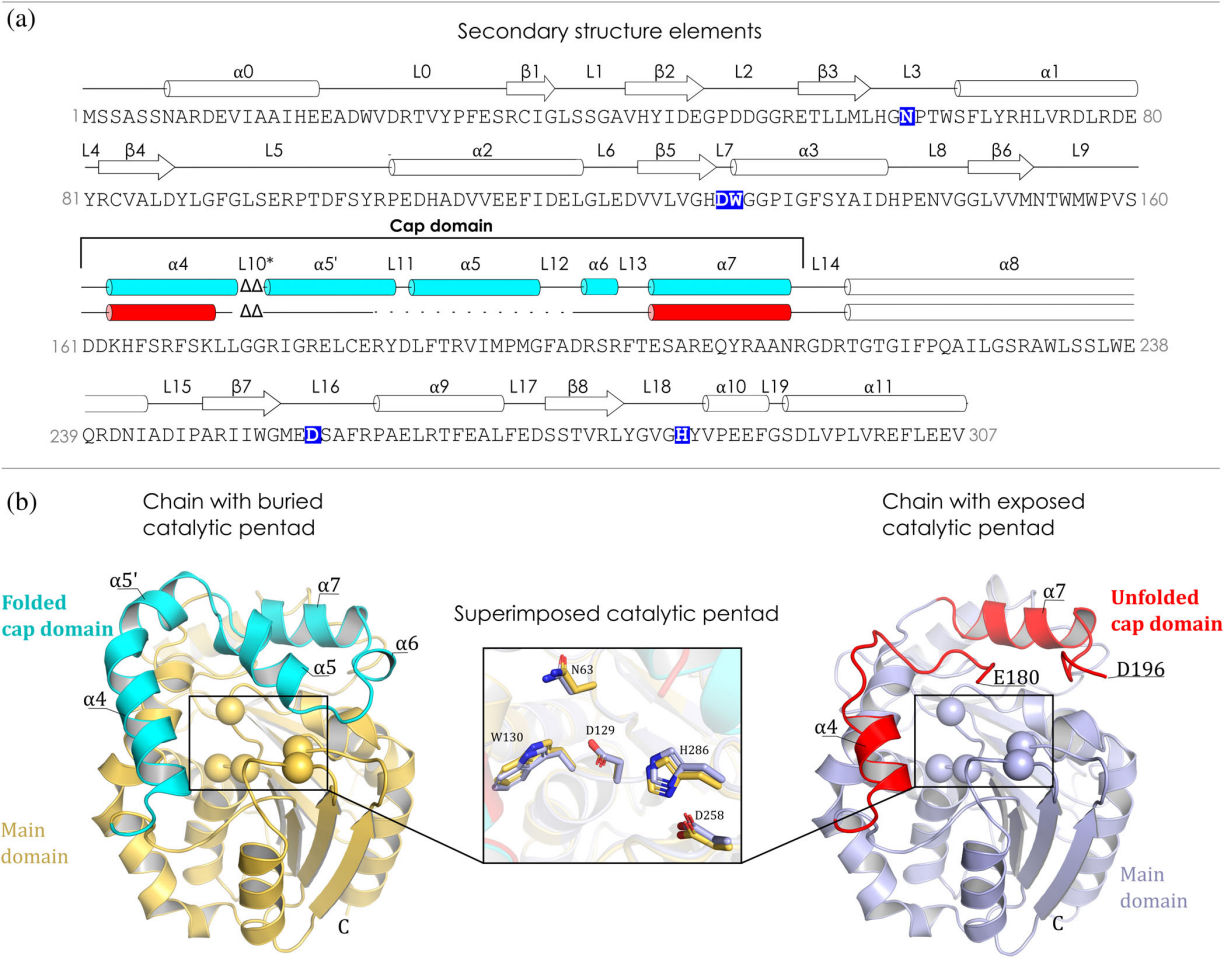
The structural features of DhmeA^ΔGG^ monomeric units. (a) The amino acid sequence of DhmeA^ΔGG^ with the secondary structure elements. The cylinders depict α‐helices, the arrows β‐strands, and the lines depict loops (L). The elements of the cap domain are colored in cyan (folded) and red (unfolded). The dotted line in the cap domain region denotes the missing residues of the incomplete cap domain. “L10*” stands for a putative loop that is not present in DhmeA^ΔGG^ variant. Catalytic residues are marked as white letters on a blue background. (b) Cartoon representation of DhmeA^ΔGG^ monomeric units. The left part of the panel depicts the chain with folded cap domain (cyan) and the catalytic pentad (spheres) being buried in the hydrophobic core of the main domain (yellow). On the right is represented the chain with unfolded cap domain (red) and the exposed catalytic pentad (spheres). The main domain is depicted in light blue. The superimposition of the catalytic pentad from both chains is presented in the middle.

A monomeric unit, or a single chain, of DhmeA^ΔGG^ canonically consists of two domains: the main α/β‐hydrolase core and the helical cap domain, with a typical organization for the HLD family (Chovancova et al., 2007). The main domain consists of eight‐stranded β‐sheets with a parallel orientation, except the antiparallel oriented β2. The β‐strands are surrounded by eight α‐helices: four on one side (α2, α3, α8, and α9) and four on the other side (α0, α1, α10, and α11). The cap domain, consisting of the residues 153–216, is formed by five α‐helices (α4, α5’, α5, α6, and α7) and five connecting loops. The protein sequence of DhmeA, accompanied by the topology of secondary structure elements, is shown in Figure 5a. The catalytic pentad of DhmeA^ΔGG^ consists of five residues, typical for the subfamily III HLDs (Chovancova et al., 2007). These residues are two halide‐binding residues (N63 and W130), a nucleophilic aspartate (D129), a catalytic acid (D258), and a histidine base (H286), as depicted in the Figure 5b.

An unusual striking feature of the DhmeA^ΔGG^ organization is that the first monomeric unit in the homodimer has a complete, properly folded helical cap domain, while the cap domain of the second monomeric unit is partially unfolded and distorted. Notably, the electron density map between residues R181 and D196, encompassing α5′ and α5 helices, is missing, and this part could not be built into the model. Due to this, a neighboring part, encompassing residues L171 and E180, is strongly distorted when compared to the canonical helical cap domain observed in the first monomeric unit. Although the distorted region can be a consequence of the introduced mutation in the cap domain, the correctly folded cap domain of the first monomer gives us confidence that the folding is not completely impaired. Therefore, we suggest that the cap domain of DhmeA protein may naturally unfold, which leads to an uncovering of the active site. As a consequence of this cap domain unfolding, the exposed catalytic machinery underneath could be accessible to bulky and large substrate molecules, which is never possible when the cap domain is fully folded.

As shown in Figure 5b, despite these structural changes in the cap domain, the positioning of catalytic pentad residues is very similar, if not identical, in both monomeric units within the homodimeric association. Moreover, the catalytic pentad residues perfectly superpose with their counterparts from DhaA and DhlA, the well‐characterized members of the subfamily I and II HLDs, respectively (Figure S1).

### 2.7 Molecular details of assembly interfaces

**FIGURE 6:**
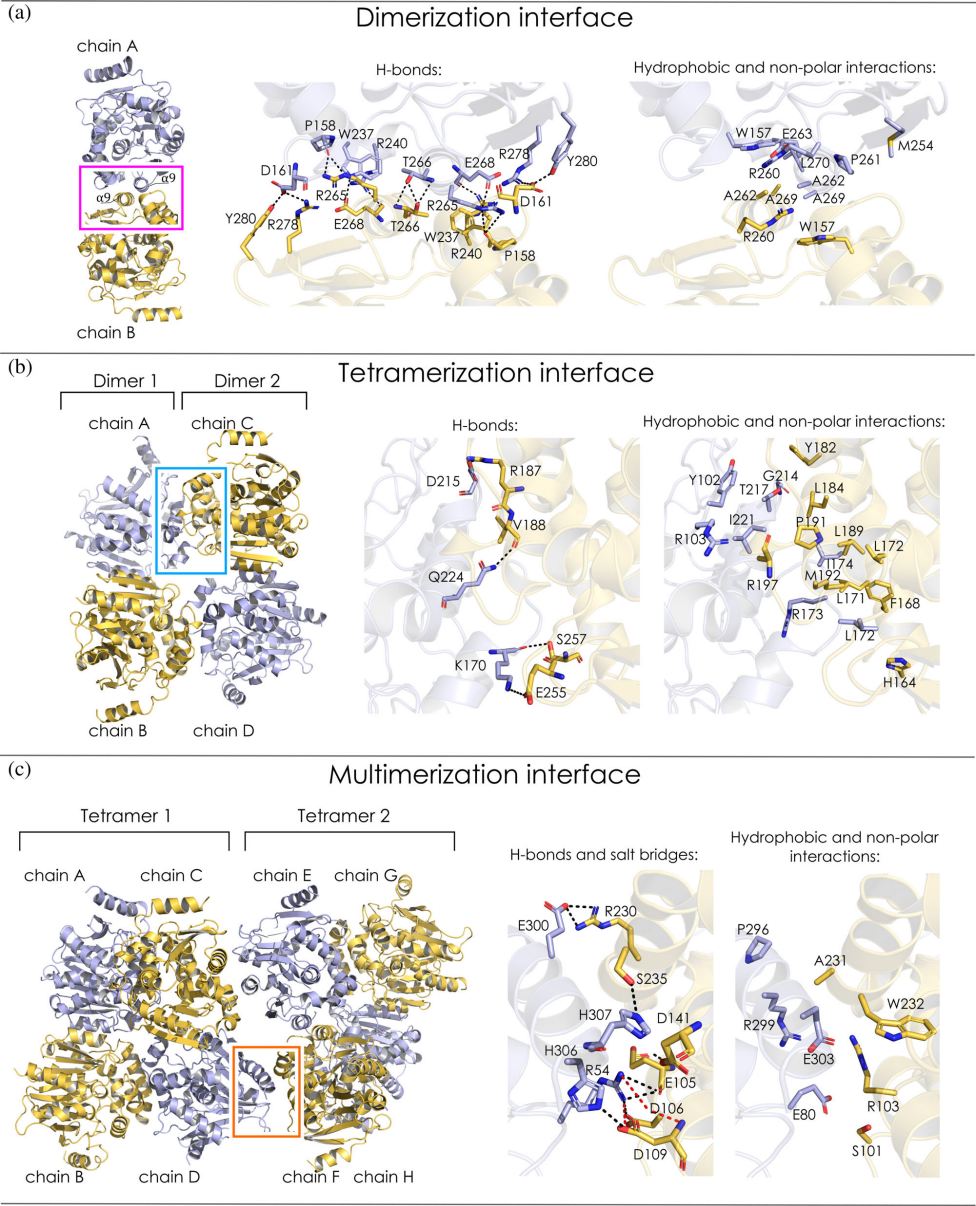
Assembly interfaces of the DhmeA^ΔGG^ crystal structure. (a) Molecular interactions at the dimerization interface between chains A and B. (b) Molecular interactions at the tetramerization interface. The tetramerization interface is formed between chains A and C and between B and D. Here, only the interface between A and C is zoomed since B and D form an analogous symmetrical interface pattern. (c) Molecular interactions at the multimerization interface. The multimerization interface is formed between chains C and E, and chains D and F. Here, only the interface between D and F is zoomed for the same reason mentioned in the panel (b). In the zoomed regions the amino acid residues involved in the interface are presented as sticks. The interfaces are formed via H‐bonds (black dashed lines), salt‐bridges (red dashed lines) and hydrophobic or non‐polar interactions.

The supramolecular ring‐like structure found in the lattice of DhmeA^ΔGG^ crystals is composed of 20 monomeric units (chains), built through homodimerization, homotetramerization, and homomultimerization interfaces (Figure 4). The dimerization interface is symmetrical, and it is located at the C‐terminal part of the main domain, encompassing α8, α9, β8, L16, and L18, although a small part of the cap domain is involved too (L9 loop). The protein–protein interactions at the dimer interface are formed mainly via H‐bonds, hydrophobic and non‐polar contacts (Figure 6a). The detailed interactions at the dimerization interface are comprehensively mapped in Supplementary Note S1.

A homotetrameric assembly of DhmeA^ΔGG^ is made by the association of two homodimers, as shown in Figure 6b. The first dimer consists of chains A and B, and the second dimer is from chains C and D. The tetramerization interface is formed between the chains A and C, and the chains B and D. The interactions between A and C chains are identical to those made by B and D chains. The tetramerization interface of DhmeA^ΔGG^ encompasses mainly the cap domain region (α4, α5’, α5, α6, and L14). The interfacial residues lying inside the main domain are part of α8, L5, and L16. The tetramerization interface is formed via H‐bonds and hydrophobic or non‐polar interactions (Figure 6b). The detailed description of molecular interactions at the tetramerization interface is provided in Supplementary Note S2.

The multimerization interface is formed between two interacting homotetrameric units (Figure 6c). The first tetramer is built from chains A to D, while the second one contains chains E to H. The oligomerization interface between these two tetramers is formed between the chains C and E, and between the chains D and F as depicted in Figure 6c, where the interactions between C and E are identical to the interactions between D and F. The multimerization interface is secured by the main α/β‐hydrolase core, where α1, α2, α3, α8, α11, L2, and L5 play important roles. The interface is formed via H‐bonds, salt bridges, hydrophobic, and non‐polar interactions with a detailed description in Supplementary Note S3. Finally, five tetramers are assembled into a supramolecular ring‐like 20‐mer structure through the multimerization interface.

The interaction interfaces were quantitatively analyzed using the PDBePISA server (Krissinel & Henrick, 2007) (Table 2). The percentage of the total solvent accessible area per chain is the highest in the case of the dimerization and the tetramerization interface (6.4% and 6.5%, respectively) followed by the multimerization interface with 4.1% coverage. The solvation free energy gain upon formation of the interface (Δ^i^G) is the most negative for the tetramerization interface, approx. −20 kcal/mol, followed by the dimerization interface with the Δ^i^G value around 0, and the multimerization interface having slightly positive Δ^i^G (around 2 kcal/mol). The *p* value of Δ^i^G for the tetramerization interface is close to 0, suggesting that no other interface of the observed area may have lower ΔG. Therefore, such an interface is a unique spot at the protein surface. The *p* values of the dimerization and the multimerization interfaces are similar, approx. 0.5, meaning that the interfaces are not “surprising” at all.

**TABLE 2:** Quantitative analysis of DhmeA^ΔGG^ assembly interfaces using the PDBePISA web server (Krissinel & Henrick, 2007).

Interface	Area (Å^2^)^a^	% of total SASA^b^	Δ^i^G (kcal/M)^c^	*p* value^d^
Dimerization	808 ± 12	6.4	−0.10 ± 0.94	0.47 ± 0.06
Tetramerization	1,650 ± 30	6.5	−20.2 ± 0.8	0.019 ± 0.006
Multimerization	1,028 ± 30	4.1	2.4 ± 1.8	0.57 ± 0.05

*Note*: The error was estimated as an average of all corresponding interfaces inside the ring‐like structure.

^a^ The average interface area.

^b^ The percentage of the total solvent accessible area per chain covered by the assembly interface.

^c^ The solvation free energy gain upon formation of the interface.

^d^ *p* Value of Δ^i^G.

### 2.8 Analysis of conservation at self‐interaction hot spots

**FIGURE 7:**
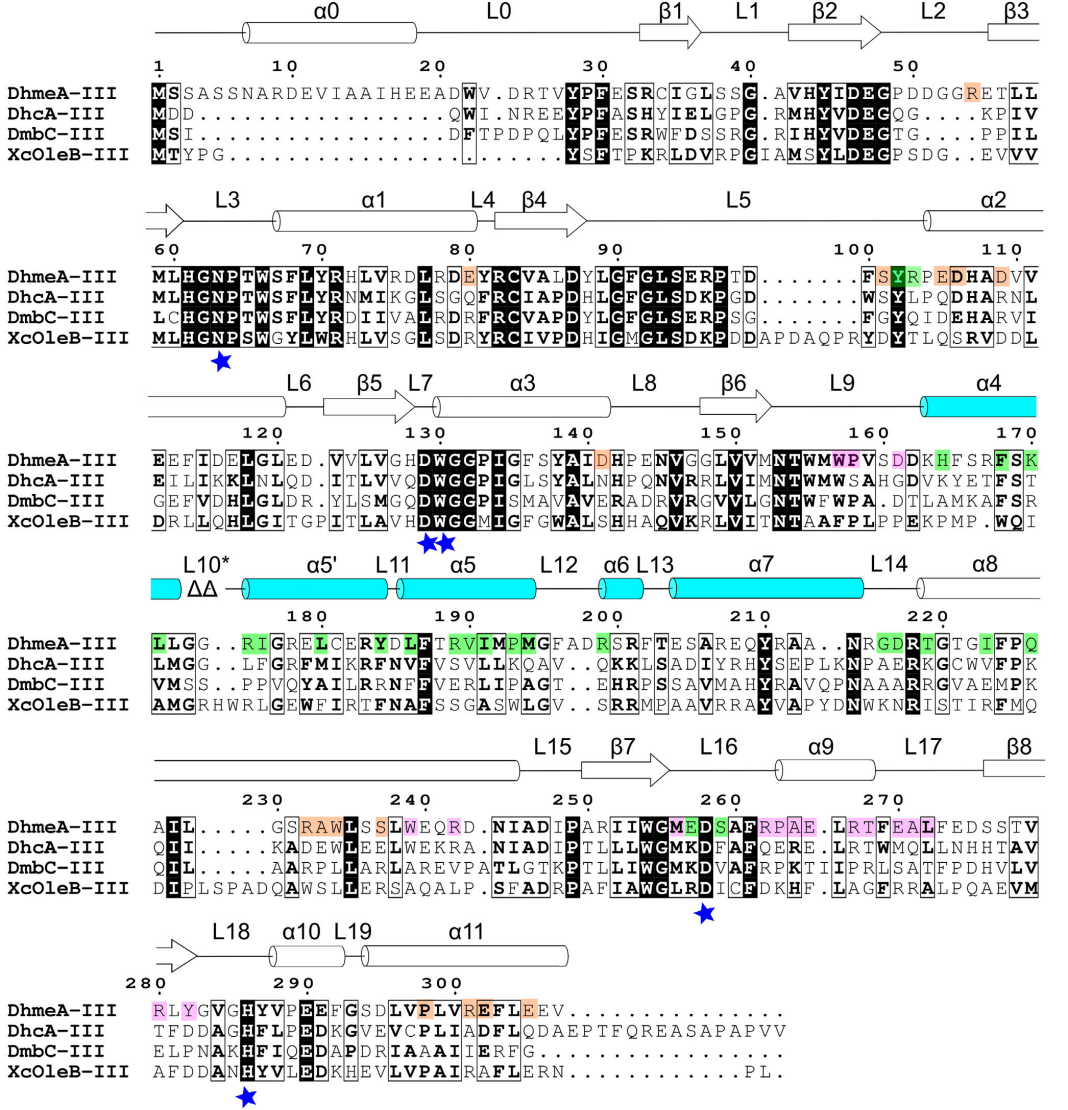
Sequence comparison between DhmeA from *Haloferax mediterranei* and its close relatives from the subfamily III HLD: DhcA from *Hahella chejuensis* KCTC, DmbC from *Mycobacterium bovis* 5033/66, and XcOleB from *Xanthomonas campestris*. The identical residues are presented as bold‐white letters on a black background, and similar residues as bold‐black letters on a white background. The secondary‐structure elements of DhmeA^ΔGG^ are shown above the sequences with the cap domain colored in cyan. The residues forming di‐, tetra‐, and multimerization interfaces are marked with the pink, green, and orange background, respectively. The catalytic residues, characteristic for subfamily III, are marked by blue stars. *L10 is not present in case of DhmeA^ΔGG^, because of the deletion of two glycines G173 and G174 (marked as ΔΔ). The alignment was generated with MAFFT (Katoh & Standley, 2013) and visualized using ESPript 3.0 (Robert & Gouet, 2014).

Here we explored the conservation level of residues responsible for the multimerization of the DhmeA protein. To this end, we generated a multiple sequence alignment between DhmeA and its close relatives from the subfamily III HLD, including DhcA from *Hahella chejuensis* KCTC, DmbC from *Mycobacterium bovis* 5033/66, and XcOleB from *X. campestris*. As shown in Figure 7, the catalytic pentad residues are conserved in the subfamily III, while the sequence encompassing the cap domain, the protein part that is responsible for the substrate specificity, is less conserved than the main α/β‐hydrolase domain. Although it has been shown experimentally that the relatives from subfamily III also form oligomeric and multimeric assemblies (Jesenska et al., 2009), their assembly interfaces are not known. Therefore, we focused solely on the conservation of the assembly residues in DhmeA^ΔGG^. The residues of the dimerization interface in DhmeA^ΔGG^ are not conserved, however, four of them (W157, P158, M256, and L172) have similar physicochemical properties compared to the aligned residues from the other three proteins. The tetramerization interface has one conserved residue (Y102) and several residues with similar physicochemical properties (F168, L171, L180, Y184, L186, I191, and M194). The multimerization interface is not conserved, but three residues have similar physicochemical properties (D106, P298, and E302).

The sequence and structure of DhmeA were also compared with members of subfamily II whose dimerization interfaces have been already reported (DbjA from *B. japonicum* (Prokop et al., 2010), DbeA from *Bradyrhizobium elkanii* (Chaloupkova et al., 2014), HanR from *Rhodobacteraceae* sp. (Novak et al., 2014), and DmxA from *Marinobacter* sp. (Chrast et al., 2019) and with DpaA from *Paraglaciecola agarilytica* NO2 (Mazur et al., 2021), a member of subfamily I, forming a tetramer (a non‐typical behavior for a HLD‐I member) (Figure S2). The tetramerization interface of DhmeA^ΔGG^ partially overlaps with the dimerization interface of DpaA in one region (conserved residues: I191 and P193) and vice versa, the dimerization interface of DhmeA^ΔGG^ partially overlaps with the tetramerization interface of DpaA in another region (conserved residues: M256 and A264). However, the dimerization interface of DhmeA^ΔGG^ does not overlap with the other members of subfamily II. The residues at the multimerization interface of DhmeA^ΔGG^ are not conserved between subfamily I and II HLDs.

In addition, we employed HotSpot Wizard (Sumbalova et al., 2018) for a prediction of the putative mutations that might have happened during the evolution of DhmeA. In total, 28 mutation hot spots were identified in DhmeA (Table S2). However, only two of them participate in the interface formation: T266 and D106, forming dimerization and multimerization interfaces, respectively. The observations from the multiple sequence alignments and HotSpot Wizard suggest that the self‐associating interfaces of DhmeA are poorly conserved and they are difficult to predict based solely on the amino acid sequence.

### 2.9 Electrostatic properties, cap domain unfolding, and consequence for catalysis

**FIGURE 8:**
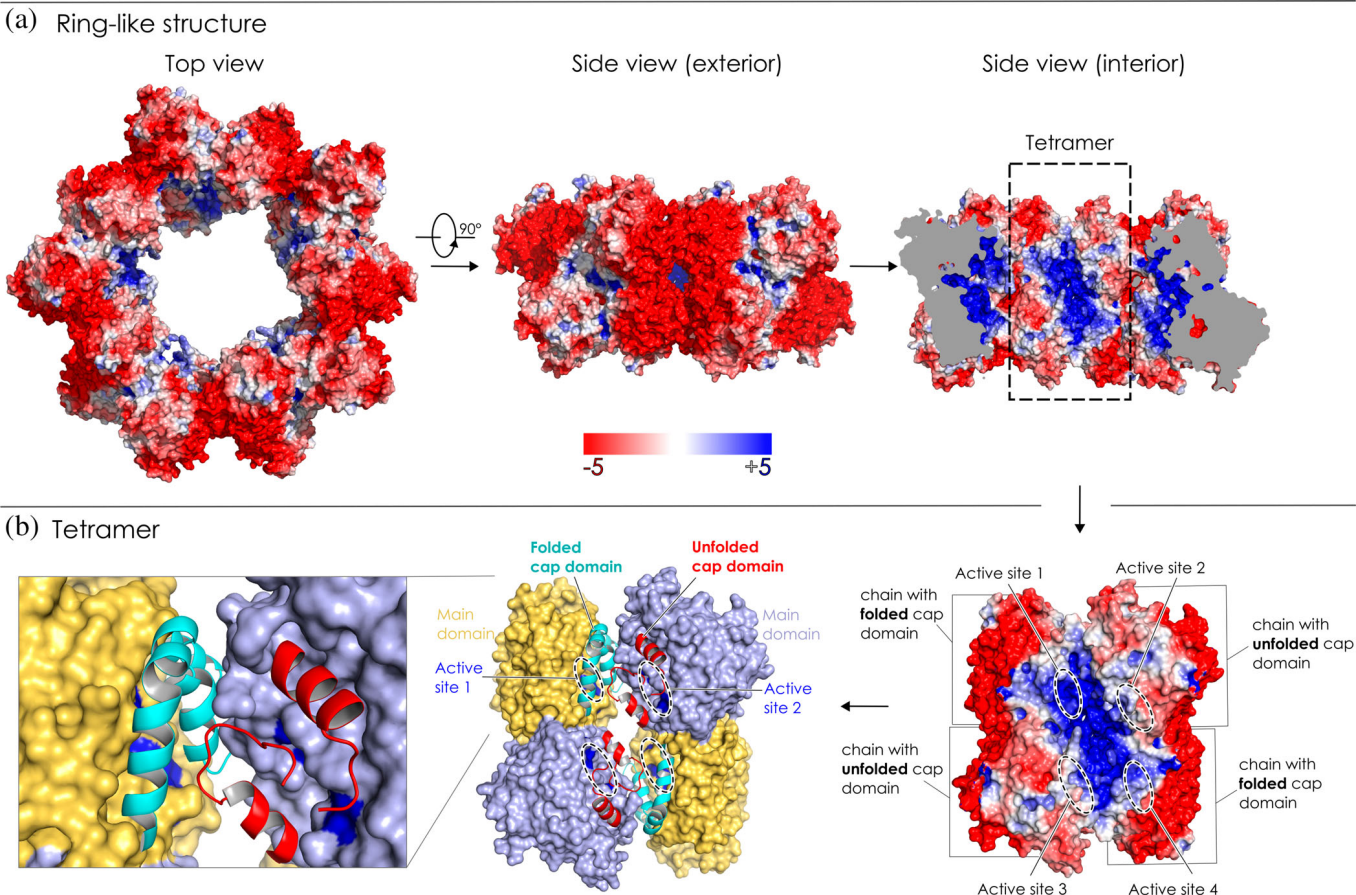
(a) Surface charge distribution of DhmeA^ΔGG^. Ring structure from the top (left panel) and from the side (exterior of the ring—middle panel and interior of the ring—right panel). Red color denotes negative charge and blue, positive charge. (b) Tetramer of DhmeA^ΔGG^ as seen from interior of the ring. Right panel: Surface charge distribution. Middle panel: Tetramer with the cap domain depicted as a cartoon (cyan for folded and red for unfolded cap domain) and main domain as a surface (yellow for a chain with folded and light blue for a chain with unfolded cap domain). Dark blue surface represents the catalytic pentad. Left panel: the zoomed view of two cap domains and catalytic pentad.

We were surprised when we displayed electrostatic potential maps on the surface of the DhmeA^ΔGG^ structure. The monomeric unit of DhmeA^ΔGG^ shows an extremely negative surface charge distribution on one side, while the other side displays a positively charged surface (Figure S3). The comparison of the DhmeA^ΔGG^ monomer with other HLDs shows a unique surface‐charge pattern for DhmeA^ΔGG^ (Figure S3). This intriguing feature becomes even more obvious in the context of the ring‐like 20‐mer structure. The exterior of the ring together with the multimerization interface has a highly negatively charged surface, while the interior is decorated with a positive charge with the highest positive surface charge density around the tetramerization interface (Figure 8a).

The anatomy and distribution of charges of the DhmeA^ΔGG^ 20‐mer ring show a certain resemblance to supramolecular ring‐like structures of some nucleic‐acid‐modifying enzymes or proteins that bind DNA, RNA, or nucleotides. As shown in Figure S4, the surface charge distributions of ring‐like structures of two recombinases, RadA from *Pyrococcus furiosus* (PDB ID: 1pzn^43^) and human Dmc1 (PDB ID: 2zjb^44^) display similar patterns. Additionally, the multiple sequence alignment of DhmeA with the archaeal recombinases (RadA from *Haloferax volcanii* [RadA_hv], RadA from *Pyrococcus furiosus* [RadA_pf], and RadA from *Saccharolobus solfataricus* P2 [RadA_ss]) shows that approximately 15% of all DhmeA residues are conserved or have similar physicochemical properties (Figure S5).

Taken together, the crystal structure of DhmeA^ΔGG^ reveals two intriguing features: (i) the mode of self‐interactions secures the positively charged enzymatic pocket (Figure 8a), and (ii) the partial unfolding of the helical cap domain exposes the catalytic machinery to the solvent (Figure 8b). Our results thus suggest that DhmeA could accommodate and catalyze processing of negatively charged large substrate molecules, such as for example nucleic acids or their precursors, however, this is only our hypothesis with no actual evidence.

## 3 DISCUSSION

The members of subfamily III are considered outliers within the HLD enzyme family, based on the following aspects: (i) their sequence annotation is low, (ii) their dehalogenase enzymatic activity is generally low, and (iii) they often form higher‐ordered quaternary structures (Vanacek et al., 2018). The mode and purpose of protein multimerization of the subfamily III HLDs are still under debate (Christenson, Jensen, et al., 2017; Christenson, Robinson, et al., 2017; Fung et al., 2015; Vanacek et al., 2018; Vasina et al., 2022). For example, XcOleB enzyme, a subfamily III member from *X. campestris*, has been shown to retain a low‐level dehalogenase activity, but it predominantly works as a β‐lactone decarboxylase within large multi‐enzyme assemblies synthesizing long‐chain olefinic hydrocarbons (Christenson, Jensen, et al., 2017; Christenson, Robinson, et al., 2017). In general, most proteins function as homodimers and/or higher‐ordered homo‐ or hetero‐assemblies. Only around one‐quarter of all human enzymes are monomeric (Chang et al., 2021; Hochberg et al., 2018). Protein multimerization may have some advantageous effects, among others, for instance, a decrease of surface‐to‐volume ratio, an increase of protein stability by reducing internal motions, a reduction of denaturation propensity and promiscuous interactions, a decrease of aggregation propensity by blocking aggregation‐prone hot‐spot regions, or enabling cooperativity (Gwyther et al., 2019; Kiss‐Szeman et al., 2022; Lynch, 2013; Marianayagam et al., 2004). East and co‐workers showed that monomeric variants of cyclohexadienyl dehydratase exhibit lower catalytic activity compared to trimeric variants, due to conformational restriction, which forces the chains to sample more compact Michaelis conformations in the trimeric form. Some of the variants even exhibited the equilibrium between the dimers and trimers, and the equilibrium was shifted by a small number of mutations at the trimer‐forming interfaces (East et al., 2022).

In this work, we probed the structural organization of a prone‐to‐multimerize DhmeA, a member of the subfamily III HLDs from the halophilic archaeon *H. mediterranei*. To date, the subfamily III is structurally poorly explored as its members have complex quaternary structures. Yet, it has been reported that a homotetrameric association can be a common feature of this multimerization (Christenson, Jensen, et al., 2017; Christenson, Robinson, et al., 2017; Fung et al., 2015). A subfamily III DmrB enzyme from *Mycobacterium* strain JS60 could be previously crystallized, but its crystals diffracted poorly (~8 Å), preventing its high‐resolution structural characterization (Fung et al., 2015). Our experiments with DhmeA from *H*. *mediterranei* showed that wild‐type DhmeA self‐associates into diverse protein quaternary structures, ranging from small oligomers to large supramolecular ring‐like assemblies of various sizes and symmetries. The most intriguing is the latter feature. The formation of macromolecular assemblies displaying various symmetries suggests the existence of a flexible protein element that can structurally re‐arrange, enabling self‐interactions in diverse symmetric modes. Recently, we demonstrated that the cap domain is the most flexible and malleable part of HLD‐fold proteins (Markova et al., 2021; Schenkmayerova et al., 2023). We, therefore, hypothesized that the cap domain of DhmeA could be this conformationally rich enigmatic element. Indeed, this hypothesis was verified by the construction of the DhmeA^ΔGG^ mutant, where we removed two glycine residues from the solvent‐exposed L10 loop. While crystallization experiments with the‐wild type DhmeA have been shown to be challenging, the DhmeA^ΔGG^ protein crystallized reproducibly and provided diffraction‐quality crystals. However, the crystals diffracted poorly (up to 3.3 Å) and the unit cell was large (*a* = 172.76 Å, *b* = 289.9 Å, *c* = 168.31 Å; α = β = γ = 90°), which complicated structure determination by molecular replacement. Key in this process was a combination of cryo‐EM data and the use of molecular modeling and advanced molecular dynamics simulations implemented in ISOLDE (Croll, 2018). The low‐resolution cryo‐EM reconstructions (~7 Å) allowed us to find orientations of two protomers in the homodimeric unit that was then successfully used for comprehensive multi‐cycle molecular replacement searches, to locate all 10 DhmeA^ΔGG^ molecules in the asymmetric unit. The crystal structure of DhmeA^ΔGG^ reveals a mode of its multimerization, where a homodimeric unit is a key repeating building block. The two homodimers self‐interact to form a homotetrameric association, and then five homotetramers assemble into a 20‐mer ring‐like structure. Note, this sequential order of the assembling presented here might not truly represent the assembly order *in vivo*.

The DhmeA protein is encoded by *H. mediterranei*, the halophilic archaeon that inhabits environments with high salt (1–4 M) concentrations (Cui et al., 2017; Han et al., 2012; Oren & Hallsworth, 2014). Therefore, the cellular components of the halophilic archaeon must be adapted to extremely high salt concentrations. It has been shown that halophilic proteins have adapted to these extremes by exhibiting the negatively charged amino‐acid residues on their surface, which allows the binding of water and salt to build up a hydrated network on the surface of the proteins (Lanyi, 1974). Structural studies have shown that the binding of the hydrated cations (provided by the excess of salt and water molecules) around the negatively charged residues on the protein surface reduces the electrostatic repulsion. At lower concentrations of salt, the protective effect is lost, and the repulsive forces between the acidic residues lead to the unfolding and the inactivation of the protein (Lanyi, 1974). Here we demonstrate that the halophilic DhmeA is no exception. The expression trials with DhmeA showed that the solubility and yield are positively correlated with increasing ionic strength of the purification buffer.

The exterior of DhmeA^ΔGG^ 20‐mer ring‐like structure is negatively charged, while its interior is decorated with a positive charge. Notably, the structural organization of the DhmeA^ΔGG^ supramolecular ring shows a resemblance to enzymatic ring‐like assemblies that bind DNA and/or nucleotides, such as DNA recombinases (Hikiba et al., 2008; Shin et al., 2003). Recently, a supramolecular ring‐like assembly of RadA‐RadB DNA recombinase complex from *Haloferax volcanii*, a moderate halophilic archaeon, was visualized by transmission electron microscopy (Patoli et al., 2017). However, its atomic‐level structure is not known yet. Our structural observations suggest that multimeric DhmeA could be involved in catalytic modifications of large, negatively charged substrate molecules (DNA, RNA, or nucleotides), although there is no experimental evidence for this. We know very little about the halogenation/dehalogenation processes of DNA (RNA) molecules in halophilic organisms. However, it is known that the halogenation of pyrimidines in DNA occurs even in non‐halophilic cells, e.g., human cells, during peroxidase‐mediated inflammatory processes (Henderson et al., 2001, 2003; Valinluck et al., 2005). Therefore, we anticipate that there might exist in many organisms a need for some halogenation enzymatic erasers.

The structure of DhmeA solved in this work is the first experimentally determined structure of any subfamily III HLD enzyme at atomic resolution. We believe that the strategy presented herein for structural characterization of multimerization‐prone protein, combining cryo‐EM and x‐ray crystallography, will serve as a template for future structural studies.

## 4 CONCLUSIONS

In this work, the structural organization of DhmeA, a subfamily III HLD‐like enzyme from the archaeon *H. mediterranei*, was determined through a combination of single‐particle cryo‐EM and x‐ray crystallography. After optimization of sample preparation steps, three‐dimensional reconstructions of oligomeric species were acquired with ~7 Å resolution by single‐particle cryo‐EM. Due to the low‐resolution gained by cryo‐EM, we engineered a crystallizable DhmeA mutant (DhmeA^ΔGG^). The crystal structure was determined due to the availability of cryo‐EM reconstruction, which allowed us to place two protomers in the homodimeric unit that was then applied in multi‐cycle molecular replacement searches, to locate all protomers in a large asymmetric unit. The 3.3 Å crystal structure revealed that DhmeA^ΔGG^ forms a ring‐like 20‐mer structure, with an outer and inner diameters of ~200 and ~80 Å, respectively. The supramolecular ring displays a negatively charged exterior, while its interior part harboring catalytic sites was positively charged. Localization and exposure of catalytic machinery suggest a processing of large, negatively charged macromolecular substrates.

## 5 MATERIALS AND METHODS

### 5.1 Molecular cloning and mutagenesis

For cryo‐EM studies, two expression vectors including full‐length (wild‐type) *dhmeA* were generated. The *dhmeA* sequence was cloned between the *Nde*I and *BamH*I restriction sites of pET21b (Sigma‐Aldrich, 69741) and pnEA/3CH^56^ vectors. The pET21b::*dhmeA* codes the protein tagged by a carboxy‐terminal hexahistidine (His_6_) tag, while the protein expressed from the pnEA/th3CH‐*dhmeA* codes the wild‐type DhmeA enzyme with an attached 3C protease cleavage site, followed by a His_6_ tag sequence, at its C‐terminal end. The latter plasmid, pnEA/th3CH‐dhmeA, was used to produce the wild‐type DhmeA for cryoEM analyses.

To stabilize DhmeA for a crystallographic study, a sequence coding two glycine residues (G173 and G174) was deleted from a putative solvent‐exposed loop L10 in the *dhmeA* sequence. This was achieved via two‐step fusion PCR‐based mutagenesis, using two oligonucleotides shown in Table S3. The resulting PCR product *dhmeA* ^*ΔGG*^ was cloned into the pET21b plasmid between the *Nde*I and *BamH*I restriction sites. It is important to note that the mutant *dhmeA* vector also contained the sequence coding for the C‐terminal poly‐histidine (His_6_) affinity purification tag. The final, error‐free expression plasmid pET21b::*dhmeA* ^*ΔGG*^ was verified by DNA sequencing.

### 5.2 Mini‐scale protein expression assays

The chemo‐competent *Escherichia coli* BL21(DE3) cells (New England Biolabs, USA) were transformed with pnEA/3CH‐*dhmeA* by heat shock. Transformed bacteria were seeded in a 6‐well plate with Luria‐Bertani (LB; Sigma‐Aldrich, USA) agar supplemented with 100 μg/mL ampicillin. The plates were incubated overnight at 37°C. Ampicillin‐resistant clones were selected and inoculated into a 24‐well plate containing 2 mL of 2 × LB (40 g LB/L), 0.5% glucose, and 100 μg/mL ampicillin. After 6 hours of incubation at 37°C and 275 rpm, the expression of the recombinant protein was induced by the addition of 2 mL of 2 × LB medium with 0.6% lactose, 0.5 mM IPTG, 20 mM HEPES with pH 7.4, and 100 μg/mL ampicillin. Plates were subsequently incubated at 22°C overnight (275 rpm). Cells were harvested by centrifugation (3700 g, 10 min) and re‐suspended in a lysis buffer (10 mM Tris–HCl with pH 8.0, 5 mM imidazole) with various concentrations of NaCl: 0.05, 0.20, 0.40, 0.60, 1.00 or 2.00 M. The re‐suspended cultures were lysed by sonication, and the lysates were centrifuged (20,000 g, 20 min). The supernatants were loaded onto separate 25 μL Talon Superflow Metal Affinity Resins (Clontech, USA), pre‐equilibrated in the respective lysis buffers. The samples were subsequently incubated on a roller shaker (4°C, 2 h). After incubation, each resin‐bound sample was washed twice with the buffer volume 1.2 mL during the centrifugation (130 g, 2 min) with the corresponding lysis buffer. The resins were re‐suspended in 40 μL of Laemmli buffer (50 mM Tris–HCl at pH 6.8, 2% SDS, 4% β‐mercaptoethanol, 30% glycerol, 0.001% Coomassie Brilliant Blue R250), and the eluted proteins were analyzed by sodium dodecyl sulfate polyacrylamide gel electrophoresis (SDS‐PAGE).

### 5.3 Protein overproduction and unoptimized protein purification

The chemo‐competent E. coli BL21(DE3) cells were transformed with the pET21b::dhmeA expression plasmid using heat shock. A single ampicillin‐resistant colony was selected and inoculated into 10 mL of LB medium (100 μg/mL ampicillin). The preculture grew 6–8 h (37°C, 150 rpm). Prior to the cultivation the EnPresso B medium (BioSilta, Finland) was carefully prepared and pre‐tempered based on manufactural protocol: five tablets of EnPresso B medium were added into 250 mL of sterile Milli‐Q water in an Ultra Yield Flask (Ukkonen et al., 2011) and dissolved. Then ampicillin (in final concentration 100 μg/mL), antifoam agent Struktol SB2020 and 125 μL of the Reagent A (glucoamylase) were added. Finally, 10 mL of preculture was used to initialize cultivation (30°C, 250 rpm). After 15–18 h cultivation, five booster tablets were added, together with 375 μL of Reagent A and 125 μL of IPTG. After 24 h of expression, cells were harvested using centrifugation (3700 g, 10 min, 4°C) and washed by 20 mM phosphate buffer (pH 7.5) with 10% glycerol. The pellets were resuspended in purification buffer A (20 mM potassium phosphate, 20 mM imidazole, 0.5 M NaCl, pH 7.5), then homogenized by the sonication and finally centrifuged (20,000 g, 1 h, 4°C) to separate DhmeA from insoluble proteins and cell debris.

The supernatant of wild‐type DhmeA enzyme with the His_6_ tag was purified in a HiTrap HP chelating column charged by Co^2+^ (Qiagen, Germany), pre‐equilibrated by the purification buffer A, and attached to the FPLC purification system (Bio‐Rad, USA). The protein was eluted from the column by purification buffer B (20 mM potassium phosphate, 500 mM imidazole, 0.5 M NaCl, pH 7.5) using a combination of linear‐gradient (0%–30% of purification buffer B) followed by a gradient with isocratic steps (30%, 60%, and 100% of purification buffer B). The target protein was eluted in 60% of buffer B, which corresponds to 300 mM imidazole.

Finally, the protein samples were concentrated and dialyzed against Tris–HCl buffer (10 mM, pH 8) overnight. The molecular weight and homogeneity of collected fractions were further assessed by SDS‐PAGE.

### 5.4 Optimized preparation of wild‐type DhmeA for cryo‐EM

*E. coli* BL21(DE3) cells were transformed by the heat shock method with the plasmid pnEA/3CH‐*dhmeA*. Ampicillin‐resistant colonies were inoculated in 10 mL of LB medium (1xLB medium, ampicillin 100 μg/mL), which was incubated (200 rpm) overnight at 37°C. The next day, the bacterial culture was used to inoculate 5‐L Erlenmeyer flasks containing 1 L of 2 × LB medium with ampicillin (100 μg/mL) where cells were grown (200 rpm, 37°C) until the culture reached OD_600_ = 0.8–1.2. Induction of expression was done at 22°C by adding 0.5 mL of 1 M IPTG, and the culture was then incubated overnight (typically 12–16 h) at 22°C and 200 rpm. The next day, the bacterial biomass was harvested by centrifugation (3,600 g, 15 min, 4°C) and re‐suspended in a lysis buffer (1 M NaCl, 10 mM Tris–HCl pH 8.0) and lysed by sonication (50% amplitude, 6 min) using a sonicator Ultrasonic Processor Hielscher UP200S (Germany). The sonicated lysate was clarified by centrifugation (20,000 g, 55 min, 4°C). The supernatant was loaded onto Talon Superflow Metal Affinity Resin (Clontech, USA) pre‐equilibrated with the lysis buffer. The His_6_‐tagged DhmeA protein was released from the Talon resin by overnight treatment with home‐made 3C protease (Marek et al., 2021) (60 μg/mL), and subsequently loaded onto a 16/60 Superdex 200 gel filtration column (GE Healthcare, UK) pre‐equilibrated with the lysis buffer supplemented with 0.5 mM TCEP (Hampton, USA). The recombinant DhmeA protein was concentrated with an *Amicon* Ultra centrifugal filter unit (Millipore, USA), and protein concentration was assayed by a DS‐11 Spectrophotometer (DeNovix, USA).

### 5.5 Optimized preparation of DhmeA^ΔGG^ for crystallization

*E. coli* BL21(DE3) cells were transformed with the recombinant plasmid pET21b::*dhmeA‐ΔGG*, plated on agar plates with ampicillin (100 μg/mL), and grown overnight at 37°C. The obtained colonies were used for the inoculation of 10 mL of LB medium with ampicillin (100 μg/mL). The cells grew overnight at 37°C and 200 rpm. The overnight culture (4–5 mL) was used to inoculate 1 L of 2 × LB medium containing ampicillin (100 μg/mL). The culture was incubated at 37°C and 150 rpm for 5–6 h, up to the point of reaching OD_600_ > 1.5. Prior to induction, the culture was cooled to 20°C for 30 min. The expression of DhmeA^ΔGG^ was induced by the addition of IPTG with a final concentration of 0.5 mM. After overnight incubation at 20°C and 150 rpm, the cells were harvested by centrifugation (20 min; 3,300 g, 4°C) and immediately resuspended in a purification buffer A (1 M NaCl, 10 mM imidazole, 10 mM Tris–HCl, pH 8). Sonication was performed to lyse the cells and the cell‐free supernatant was extracted after centrifuging at 4°C, 20,000 g for 1 hour.

The supernatant containing DhmeA^ΔGG^ was used for the purification by metallo‐affinity chromatography via a C‐terminal His_6_‐tag, using the 5 mL Ni‐NTA Superflow column (Qiagen, Germany) attached to the FPLC purification system BioLogic DuoFlow (Bio‐Rad, USA). A gradient of 0%, 10%, 60%, and 100% of purification buffer B (1 M NaCl, 500 mM imidazole, and 10 mM Tris–HCl, pH 8) was used for the elution process. The target protein was eluted by 60% of the buffer B, corresponding to 300 mM imidazole.

Protein fractions eluted at 60% of buffer B were collected for further separation by GPC. The samples were loaded onto a HiLoad™ 16/600 Superdex™ 200 column (GE Healthcare, Sweden) on an Äkta Purifier FPLC system (GE Healthcare, Sweden). The column was pre‐equilibrated by separation buffer (1 M NaCl, 0.5 mM TCEP, and 10 mM Tris–HCl, pH 8), and the constant flow rate was set at 1 mL/min. The protein eluted in two major peaks: the first peak contained high‐molecular‐weight complexes, while the second peak contained lower‐molecular‐weight complexes. The fractions containing the latter peak were collected, concentrated with an Amicon Ultra centrifugal filter unit (Merck Millipore Ltd), and re‐separated with GPC to yield a monodisperse protein sample suitable for crystallization experiments. The second GPC step was essential to obtain crystallization‐quality protein. The enzyme concentration was determined by DS‐11 Spectrophotometer (DeNovix, USA) with an extinction coefficient of DhmeA 1.73 mL (mg cm)^−1^ at 280 nm, and the product purity was validated by SDS‐PAGE.

### 5.6 Differential scanning fluorimetry

The thermal stability of the DhmeA (2 mg/mL) in a buffer containing 1 M NaCl, 10 mM Tris (pH 8.0), and 0.5 mM TCEP was analyzed by a label‐free differential scanning fluorimetry (DSF) using a Prometheus NT.48 instrument (NanoTemper Technologies, Germany). The temperature‐triggered unfolding was measured in the temperature range of 20–100°C. The measurements were carried out in nanoDSF‐grade high‐sensitivity glass capillaries (NanoTemper Technologies, Germany) at a heating rate of 1°C/min. Melting temperatures (*T*_m_) were inferred from the first derivative of the ratio of tryptophan fluorescence emission intensities at 330 and 350 nm.

### 5.7 Circular dichroism spectroscopy

Circular dichroism (CD) spectra of the DhmeA (2 mg/mL) in a buffer containing 1 M NaCl, 10 mM Tris (pH 8.0), and 0.5 mM TCEP were recorded at room temperature using a Chirascan CD Spectrometer equipped with a Peltier thermostat (Applied Photophysics, UK). CD data were expressed as mean residue ellipticity (*Θ*_MRE_).

### 5.8 Dynamic light scattering

Protein solutions were centrifuged (16,000 g, 10 min) prior to dynamic light scattering (DLS) measurement to remove the impurities. DLS experiments were conducted with wild‐type DhmeA solution (1 mg/mL) in a buffer containing 1 M NaCl, 10 mM Tris (pH 8.0), and 0.5 mM TCEP, using the Delsa Max Core (Beckman Coulter, USA).

### 5.9 Cryo‐EM data acquisition

The full‐length DhmeA (3.5 μL, 0.035 mg/mL) was applied to freshly glow‐discharged (Quorum, SC7620 Sputter Coater) TEM grids (Quantifoil, Cu, 200 mesh, R2/1) and vitrified in liquid ethane using a Vitrobot mark IV (Thermo Scientific) with single blotting, 15 s wait time, −2 blot force, 4.5 s blot time and no drain time. Grids were subsequently mounted into Autogrid cartridges and loaded to the FEI Tecnai F20 and Titan Krios (Thermo Fisher Scientific) transmission electron microscopes for screening and data acquisition, respectively.

Two datasets with the sample tilt of 0° and 44°, respectively, were collected for the data analysis using the Titan Krios microscope. The experimental details of the data acquisition are summarized in Table S4.

### 5.10 Cryo‐EM data analysis

Both data sets were analyzed using cryoSPARC (Punjani et al., 2017) software. MotionCor2 (Zheng et al., 2017) and Gctf (Zhang, 2016) were used for motion correction and CTF estimation as it is implemented in cryoSPARC. First, particles from 20 micrographs were manually selected and classified to generate templates for template picking (cryoSPARC). The overall number of 1,237,502 and 439,939 particles was extracted from the data collected at 0° and 44° stage tilt, respectively. After 2D classification, 81,595 particles were selected for cryoSPARC *ab initio* reconstruction with 3 models followed by multiple rounds of 3D classification (heterogeneous refinement job). Finally, 64,146 particles were processed for Non‐uniform Refinement (cryoSPARC) with a final resolution of 7 Å.

### 5.11 Preparation of structural models

Models for cryo‐EM reconstruction were prepared for the docking calculations using the same workflow as for molecular replacement. A search with HHpred (Zimmermann et al., 2018) was used to find and align the sequences of homologs of known structure. Using this alignment, the program Sculptor (Bunkoczi & Read, 2011) was used to edit the top three hits from HHpred, PDB entries 3g9x (Stsiapanava et al., 2010), 4f0j, and 4 psu, to truncate non‐identical side chains and remove unaligned portions of the structure. Then the program Ensembler (Bunkóczi & Read, 2011) was used to superimpose the three sculpted models and to trim regions deviating among the structures to leave an ensemble model of the conserved core.

As an alternative, the Robetta server was used to construct a model of DhmeA, using the trRosetta deep‐learning algorithm (Yang et al., 2020). The first 25 residues of this model were judged to be unreliable and were omitted, as the modeling assigned them expected RMS errors ranging from 2 to 12 Å.

### 5.12 Docking models into cryo‐EM reconstruction

Docking calculations were performed using the real‐space molecular replacement features of Phaser (McCoy et al., 2007) providing the Fourier terms corresponding to the cryo‐EM reconstruction as target structure factors. In this approach, the Phaser likelihood‐based rotation function is computed to obtain a set of trial orientations, then the phased translation function (Colman et al., 1976; Read & Schierbeek, 1988) finds the translation of the oriented model that maximizes its correlation with the target map.

Initial attempts to place the ensemble model into the full cryo‐EM reconstruction failed to yield a clear answer. To reduce the noise in the calculation, the program phenix.map_box (Liebschner et al., 2019) was used to extract the unique portion of the map representing a dimer. When this was used as the target map, two unambiguous placements were found, generating a dimer model. A full hexamer was then generated by applying the remaining threefold symmetry to the dimer model.

### 5.13 Optimizing the hexamer model to fit the cryo‐EM reconstruction

The trimmed ensemble comprises only the conserved core of wild‐type DhmeA, containing only 126 of the 307 residues in the full‐length protein. A more complete model could be constructed from the model derived using Sculptor from the top HHpred hit, PDB entry 3g9x, which contains 279 residues that match in the sequence alignment. The additional residues largely fit within the reconstruction. A rigid‐body optimization in ChimeraX (Goddard et al., 2018) of the correlation between one chain of the model and the cryo‐EM reconstruction gave a correlation coefficient of 0.903. However, a hexamer constructed from the trimmed trRosetta model provided an even better fit to the map, with a correlation coefficient of 0.936. Subsequent work therefore focused on the trRosetta model.

ISOLDE (Croll, 2018) was used to modify the hexamer model to optimize the fit to the cryo‐EM reconstruction. First, residues making intractable severe clashes between chains (W239 and L272) were pruned back to the beta‐carbon. Clashes between R262 and the opposing D161 were resolved by adjusting the rotamers. Overfitting was avoided by applying torsion restraints to maintain the backbone geometry of all α‐helices and β‐strands. During the fitting simulation, the temperature was gradually reduced from 100 to 0 K. The most obvious result of the fitting was an increase in the spacing between monomers by about 2.7 Å (centroid‐centroid distance), combined with subtle changes to the relative positions of secondary structure elements.

### 5.14 Crystallization of DhmeA^ΔGG^ enzyme

A freshly purified sample of DhmeA^ΔGG^ was concentrated to 12–14 mg/mL and set up for crystallization in 24‐well crystallization plates (Hampton Research, USA) at 10°C using the sitting‐drop vapor‐diffusion method. The crystallization mother liquor contained 0.1 M MES/imidazole buffer system (pH 6.5), 0.1 M amino acids, 10% PEG 4,000, and 20% glycerol, originating from the Morpheus screen (Molecular Dimensions Ltd, UK). Each drop consisted of 2 μL of the enzyme and 2 μL of the mother liquor equilibrated against 500 μL of the reservoir solution. The crystal growth was monitored by an Olympus‐SZX10 (Olympus) stereomicroscope. The crystals formed within 2–5 days. The obtained crystals were fished out and cryo‐cooled in liquid nitrogen.

### 5.15 X‐ray data processing

Diffraction data were collected at the PXIII beamline at SLS Synchrotron (Villigen, CH) at a wavelength of 0.999 Å. Multiple data sets from the best diffracting crystals were processed using XDS (Kabsch, 2010) Version February 5, 2021, scaled via XSCALE, and merged by XDSCONV. The selection of datasets that were merged was guided by the program XDSCC12 (Assmann et al., 2020).

### 5.16 Molecular replacement calculations

Based on considerations of solvent content (Kantardjieff & Rupp, 2003; Matthews, 1968), up to 12 copies of the DhmeA^ΔGG^ monomer were expected in the asymmetric unit of the crystal. Molecular replacement searches with Phaser (McCoy et al., 2007) failed with all monomer models, including the individual models processed with Sculptor, the trimmed ensemble model, and the trRosetta model. This was not surprising given the relatively low resolution, the limited sequence identity of available templates, and the small fraction of the asymmetric unit content accounted for by these models. The hexamer model constructed by docking the trRosetta model into the cryo‐EM reconstruction was tested next, searching for two copies, but this also failed to yield any convincing solutions. Given the possibility that the hexamer could sit on a crystallographic 2‐fold axis in the space group C2221, a search for four copies of a trimer model was also attempted, again unsuccessfully.

It was noted that the deletion of glycine residues G173 and G174 would be located near the 3‐fold axis of the hexamer, raising the possibility that the quaternary structure had been perturbed. Therefore, a dimer extracted from the hexamer was tested as a potential molecular replacement model. This failed initially, but when a dimer was extracted from the hexamer model that had been optimized in ISOLDE, a search for six copies gave a solution in which the first five dimers were placed with excellent signal, but the placement of the sixth copy failed to increase the log‐likelihood‐gain score. Inspection of this potential solution showed that there were in fact only five dimers in the asymmetric unit of the crystal. When a sixth copy was placed, it had poor electron density and clashed badly with other chains, but a model comprising the first five dimers packed well in the unit cell. Notably, residues 173–189 (beginning with the G173 and G174 deleted in the crystallization construct and modeled by trRosetta as an α‐helix) clashed severely with adjacent dimers and were removed prior to initial rebuilding.

### 5.17 Model building and refinement

Initial rebuilding of the model was performed in ISOLDE, aided markedly by a 10‐fold NCS‐averaged map generated using Resolve density modification in Phenix (Terwilliger, 2002). Rebuilding one chain in this map, reimposing strict symmetry on the remaining chains and refining with torsion‐angle NCS restraints in phenix.refine reduced Rwork/Rfree from 0.38/0.42 to 0.32/0.35. Further inspection revealed clear differences between the two monomers in each dimer, so further rebuilding was performed in non‐averaged maps.

### 5.18 Crystal structure representation

The crystal structure of DhmeA^ΔGG^ was visualized by PyMOL (Schrödinger, LLC, 2019). Protein surface charge distribution was calculated by APBS Electrostatics (Jurrus et al., 2018) which was used as a plugin of PyMOL. The default settings of PDBePISA server (Krissinel & Henrick, 2007) were used to (i) identify the interactions at assembly interfaces and their corresponding distances, (ii) calculate the interface area, solvation free energy gain upon formation of the interface (Δ^i^G), and *p* value.

## AUTHOR CONTRIBUTIONS

Klaudia Chmelova, Tadeja Gao, Martin Polak, and Andrea Schenkmayerova contributed equally to this study. Klaudia Chmelova and Martin Marek prepared and cloned the DNA constructs and produced the protein samples for structural and biochemical experiments. Klaudia Chmelova, Radka Chaloupkova, Tanvir R. Shaikh, and Jiri Novacek performed initial cryo‐EM imaging. Martin Polak and Jiri Novacek collected and processed EM data. Klaudia Chmelova and Martin Marek carried out the crystallization screenings, and optimized crystallization hits. Andrea Schenkmayerova, Tristan I. Croll, Kay Diederichs, Randy J. Read, and Martin Marek collected diffraction data and solved the protein crystal structure. Radka Chaloupkova, Jiri Damborsky, and Martin Marek designed the project, supervised research, and interpreted data. Tadeja Gao, Jana Skarupova, Andrea Schenkmayerova, and Martin Marek wrote the manuscript with contributions from all authors. All authors have approved the final version of the manuscript.

## CONFLICT OF INTEREST STATEMENT

The authors declare no conflicts of interest.

## Supporting information


**Data S1.** Supporting Information.

## Data Availability

Atomic coordinates and structure factors have been saved in the Protein Data bank (www.wwpdb.org) (Berman et al., 2000) under PDB ID accession code: 8CKP and EMDB ID accession code: EMD‐17015. The authors will release the atomic coordinates and experimental data upon article publication.
