# Supplementary material for: Multimeric structure of a subfamily III haloalkane dehalogenase‐like enzyme solved by combination of cryo‐EM and x‐ray crystallography
Source: Protein Sci. 2023 Oct 1;32(10):e4751. doi: 10.1002/pro.4751 (PMC10503415; doi:10.1002/pro.4751)
Supplement: Supplementary file 1 — Data S1. Supporting Information. [file PRO-32-e4751-s001.docx]

**Supporting Information**

**Multimeric structure of a subfamily III haloalkane dehalogenase-like enzyme solved by combination of cryo-EM and X-ray crystallography**

Klaudia Chmelova^1,2,#^, Tadeja Gao^1,2#^, Martin Polak^3,#^, Andrea Schenkmayerova^1,2,#^, Tristan I. Croll^4^, Tanvir R. Shaikh^3,5^, Jana Skarupova^1^, Radka Chaloupkova^1,2^, Kay Diederichs^6^, Randy J. Read^4^, Jiri Damborsky^1,2^, Jiri Novacek^3^, and Martin Marek^1,2,*^

1. Loschmidt Laboratories, Department of Experimental Biology and RECETOX, Faculty of Science, Masaryk University, Kamenice 5, Bld. C13, 625 00 Brno, Czech Republic
2. International Clinical Research Center, St. Anne’s University Hospital Brno, Pekarska 53, 656 91 Brno, Czech Republic
3. Central European Institute of Technology, Masaryk University, Kamenice 753/5,
   625 00 Brno, Czech Republic
4. Department of Hematology, Cambridge Institute for Medical Research, University of Cambridge, Hills Road, Cambridge CB2 0XY, United Kingdom.
5. University Medical Center Göttingen, Institute of Neuropathology, Justus-von-Liebig-Weg 11, 37077 Göttingen, Germany
6. Department of Biology, University of Konstanz, Universitätsstrasse 19, 78457 Konstanz, Germany

^#^ These authors contributed equally to this study.

* Corresponding author: martin.marek@recetox.muni.cz

**Table S1.** Crystallographic data collection and refinement statistics.

|  | DhmeA^∆GG^ |
| --- | --- |
| Data collection |  |
| Wavelength (Å) | 0.999 |
| Space group | *C*222_1_ |
| Cell dimensions |  |
| a, b, c (Å) | 172.76, 289.9, 168.31 |
| α, β, γ (°) (°) | 90, 90, 90 |
| Resolution (Å) | 49.47 - 3.306 (3.424 - 3.306) |
| Total reflections | 4450892 (95040) |
| Unique reflections | 63328 (6044) |
| Rmerge | 0.3529 (6.506) |
| I/σI | 10.76 (0.43) |
| Completeness (%) | 99.55 (96.8) |
| Multiplicity | 70.3 (15.2) |
| CC (1/2) | 0.999 (0.241) |
| Wilson B-factor | 151.61 |
| Refinement |  |
| Resolution (Å) | 49.47 - 3.306 (3.424 - 3.306) |
| No. reflections | 63073 (6041) |
| Rwork / Rfree (%) | 23.21 / 27.5 |
| No. atoms |  |
| Protein | 23882 |
| Water | 1 |
| B-factors | 155.02 |
| Protein | 155.02 |
| Water | 148.80 |
| R.m.s. deviations |  |
| Bond lengths (Å) | 0.003 |
| Bond angles (°) | 0.73 |
| Ramachandran favored (%) | 95.55 |
| Ramachandran allowed (%) | 4.31 |
| Ramachandran outliers (%) | 0.14 |
| PDB ID code | 8CKP |

**Table S2.** Mutation hot spots of DhmeA predicted by HotSpot Wizard^1^. * and ** indicate whether the residue represents the mutagenesis hot spot based on the majority consensus and on the frequency ratio consensus approach, respectively. M and D represent multimerization and dimerization interface, respectively.

| **Residue** | **Residue No.** | **Hot spot by majority*** | **Hot spot ratio**** | **Interface** |
| --- | --- | --- | --- | --- |
| V | 27 | no | yes | no |
| G | 36 | no | yes | no |
| S | 39 | yes | yes | no |
| A | 41 | no | yes | no |
| E | 55 | yes | yes | no |
| V | 74 | yes | no | no |
| V | 84 | yes | no | no |
| D | 106 | no | yes | **M** |
| E | 117 | no | yes | no |
| H | 128 | yes | no | no |
| F | 136 | yes | yes | no |
| H | 142 | yes | no | no |
| N | 145 | yes | yes | no |
| V | 151 | yes | no | no |
| C | 179 | no | yes | no |
| D | 183 | yes | yes | no |
| T | 186 | yes | yes | no |
| S | 203 | no | yes | no |
| Q | 207 | no | yes | no |
| G | 220 | no | yes | no |
| G | 228 | no | yes | no |
| I | 243 | yes | yes | no |
| I | 246 | no | yes | no |
| R | 249 | yes | yes | no |
| T | 266 | yes | yes | **D** |
| E | 272 | no | yes | no |
| V | 282 | yes | yes | no |
| E | 289 | yes | yes | no |

**Table S3.** Nucleotide sequences of PCR oligonucleotides used in this study.

| **Name** | **Sequence (5´→3´)** | **Sense** |
| --- | --- | --- |
| dhmeA-ΔGG-Fv | CCCGTTTCTCCAAACTGCT GCGCATCGGCCGTGAACTGTGTG | Forward |
| dhmeA-His-Rv | GGGTGTAGGATCCTTAATGGTGGTGATGGTGATGTACTTCTTCCAGAAATTCGCG | Reverse |
| dhmeA-Fv | TTGTGTCATATGTCCTCCGCAAGCTCTAACG | Forward |
| dhmeA-ΔGG-Rv | CAGCAGTTTGGAGAAACGGG | Reverse |

**Table S4.** The experimental details of the cryo-EM data acquisition.

| **Sample tilt [deg]** | **0°** | **44°** |
| --- | --- | --- |
| **Microscope** | Titan Krios | Titan Krios |
| **Voltage [kV]** | 300 | 300 |
| **Camera** | Falcon 2 | K2 |
| **GIF** | NO | YES |
| **Energy slit [eV]** | --- | 10 |
| **Data acquisition software** | EPU (Thermo Scientific) | EPU |
| **Pixel size [A]** | 1.061 | 0.822 |
| **Exposure time [s]** | 1.5 | 12 |
| **Total dose [e/A^2]** | 49 | 76 |
| **Fractions** | 29 | 40 |
| **Movies in dataset** | 6281 | 2754 |


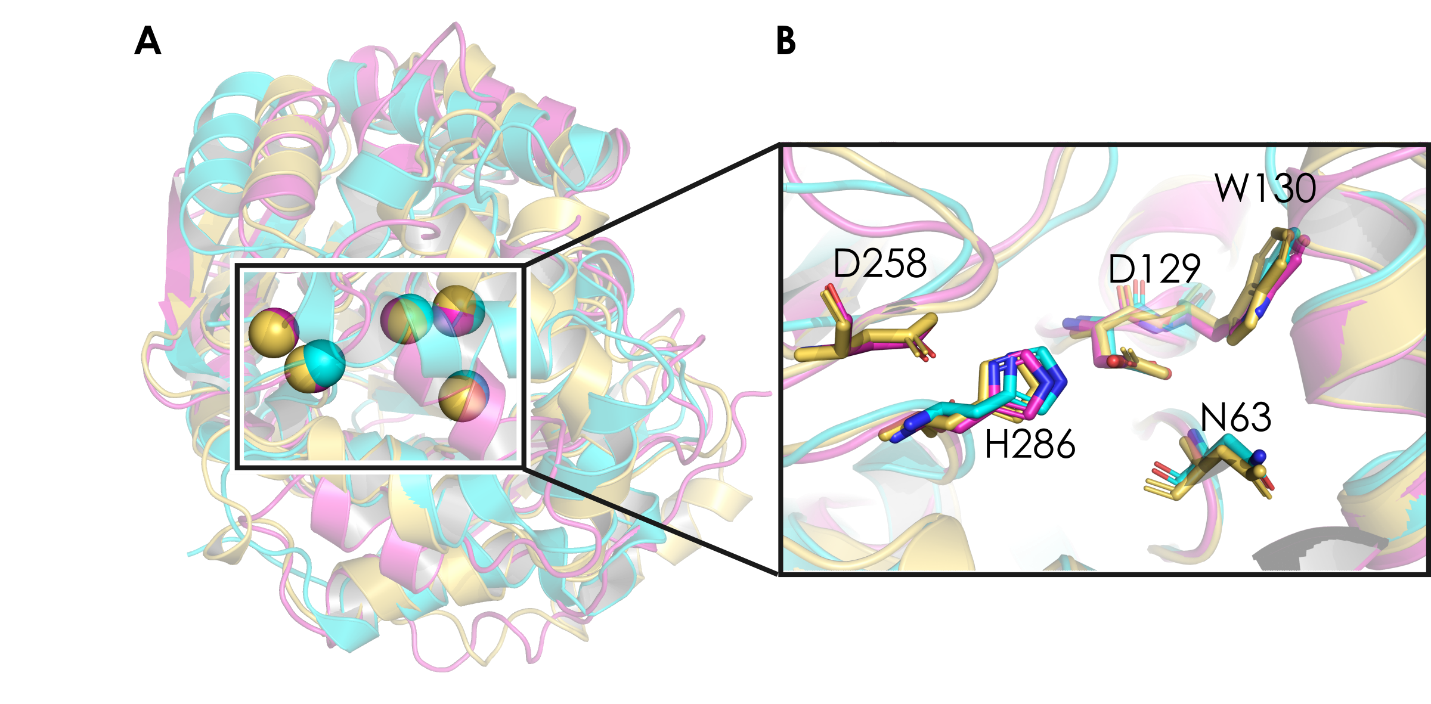


**Fig. S1.** The superposition of DhmeA^ΔGG^-III (yellow) with DhaA-II (cyan, PDB ID: 4hzg^2^) and DhlA-I (pink, PDB ID: 2dhc^3^). A: Cartoon of the monomeric units with the catalytic pentad depicted as spheres. B: Zoomed region of the catalytic pentad (sticks).


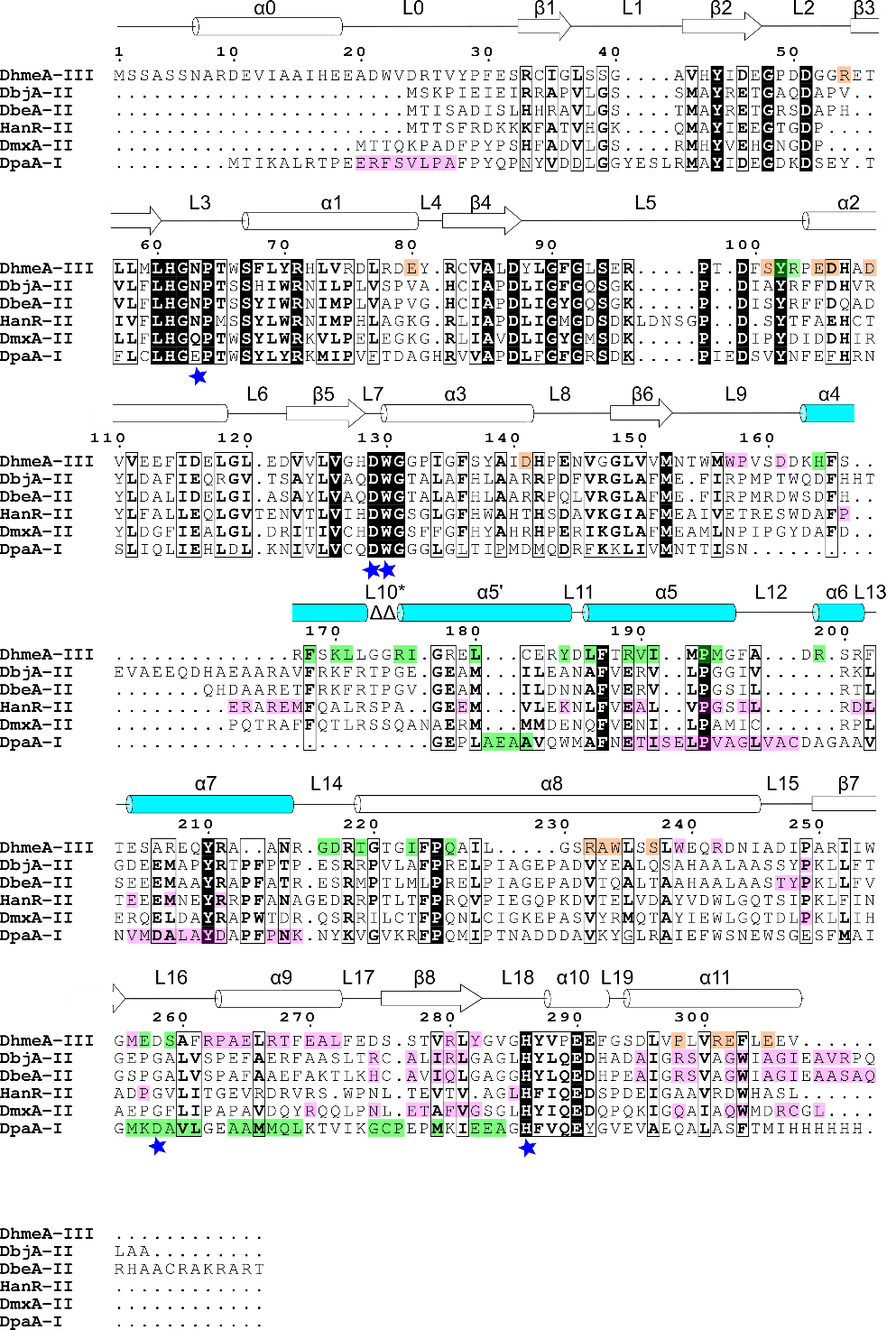


**Fig. S2.** Multiple sequence alignment of DhmeA from *Haloferax* *mediterranei*, with the members of the subfamily II: DbjA from *B. japonicum* (PDB ID: 3a2m^4^), DbeA from *Bradyrhizobium elkanii* (PDB ID: 4k2a^5^), HanR from *Rhodobacteraceae sp.* (PDB ID: 4brz^6^), and DmxA from *Marinobacter sp.* (PDB ID: 5mxp^7^). DpaA from *Paraglaciecola agarilytica* NO2 (PDB ID: 7avr^8^) is a member of the subfamily I. The identical residues are presented as bold-white letters on a black background, and similar residues as bold-black letters on a white background. The secondary-structure elements of DhmeA^ΔGG^ are shown above the sequences with the cap domain coloured in cyan. The residues forming di-, tetra-, and multimerization interfaces are marked with the pink, green, and orange background, respectively. Catalytic residues, typical for the subfamily III, are marked by the blue stars. *L10 is not present in case of DhmeA^ΔGG^, because of the double deletion of two Gly (marked as ΔΔ). The alignment was generated with MAFFT^9^ and visualized using ESPript 3.0.^10^


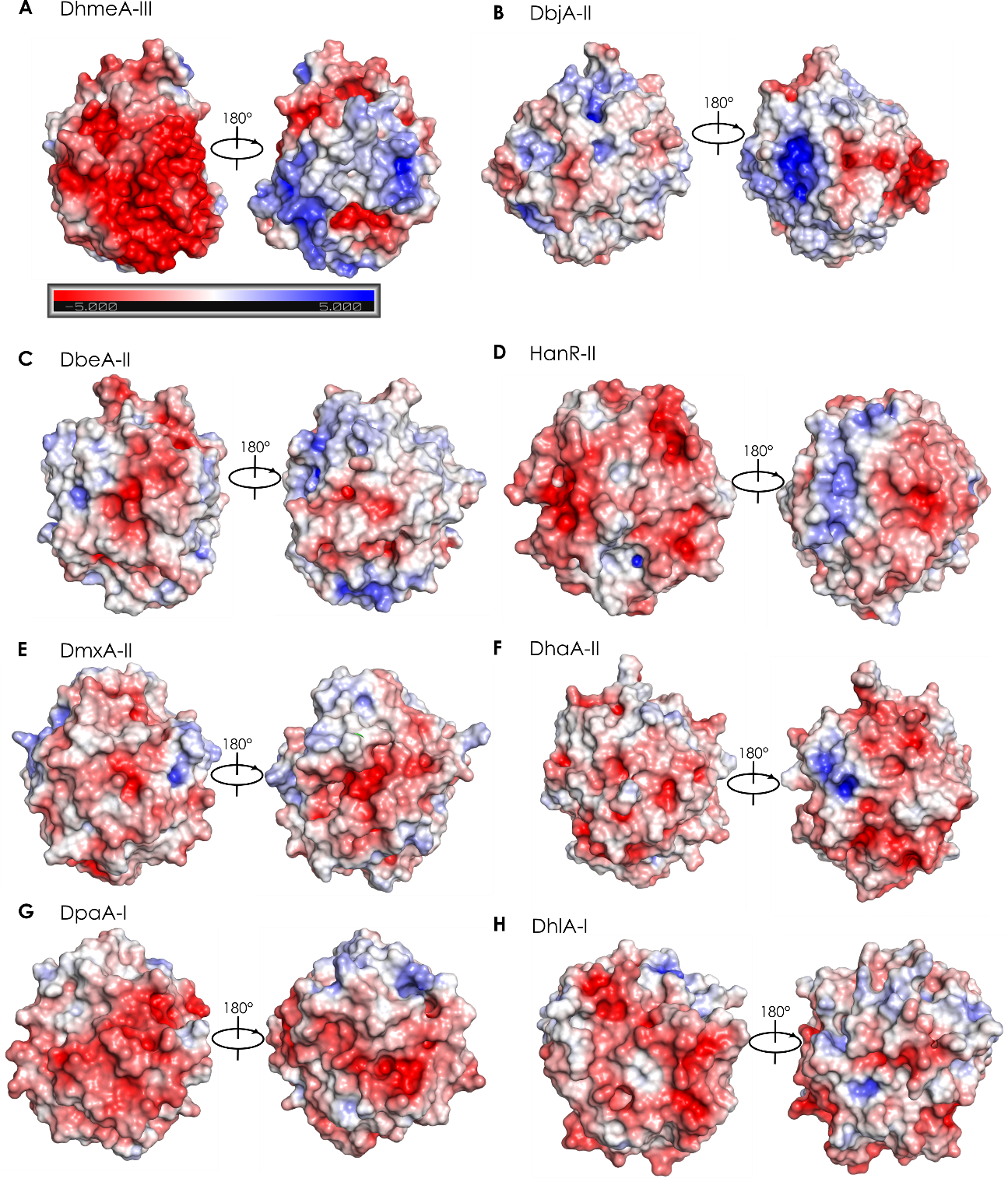


**Fig. S3.** Surface charge distribution of the monomeric unit of several representative HLDs. (A) DhmeA^ΔGG^ from *Haloferax mediterranei* is a member of subfamily III. (B) DbjA from *Bradyrhizobium japonicum* (PDB ID: 3a2m^4^), (C) DbeA from *Bradyrhizobium elkanii* (PDB ID: 4k2a^5^), (D) HanR from *Rhodobacteraceae sp.* (PDB ID: 4brz^6^), (E) DmxA from *Marinobacter sp.* (PDB ID: 5mxp^7^), (F) DhaA from *Rhodococcus rhodochrous* (PDB ID: 4hzg^2^) are the members of subfamily II. (G) DpaA from *Paraglaciecola agarilytica* NO2 (PDB ID: 7avr^8^), and (H) DhlA from *Xanthobacter autotrophicus* GJ10 (PDB ID: 2dhc^3^) are the members of subfamily I. Red colour represents negative, white zero, and blue positive charge.


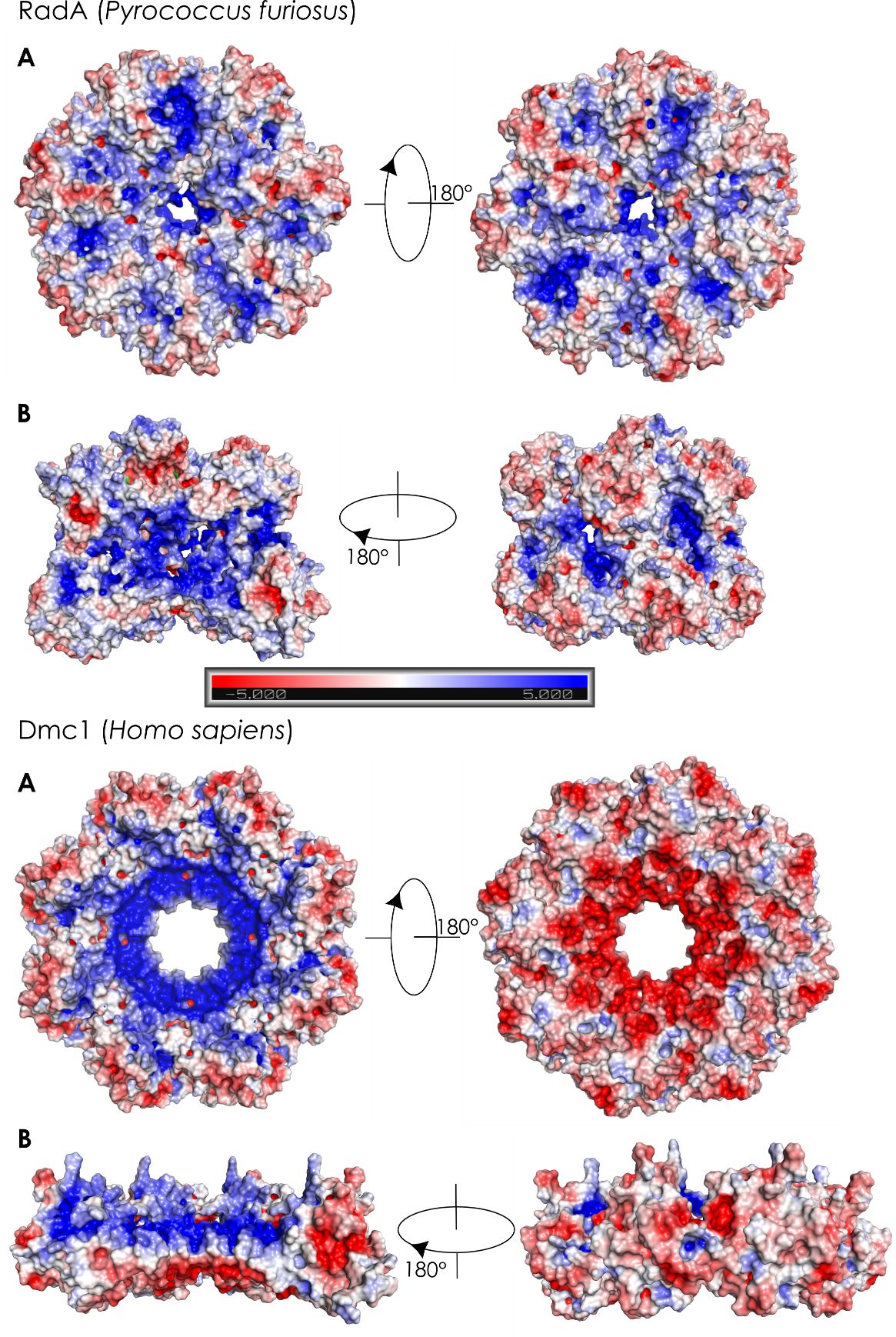


**Fig. S4.** Surface charge distribution of RadA from *Pyrococcus furiosus* (PDB ID: 1pzn^11^) on the top, and Dmc1 from *Homo sapiens* (PDB ID: 2zjb^12^) on the bottom. (A) The top view of the ring (left) and the bottom view of the ring (right). (B) The view from interior side of the ring (left), and the view from exterior side (right). Red colour represents negative, white zero, and blue positive charge.


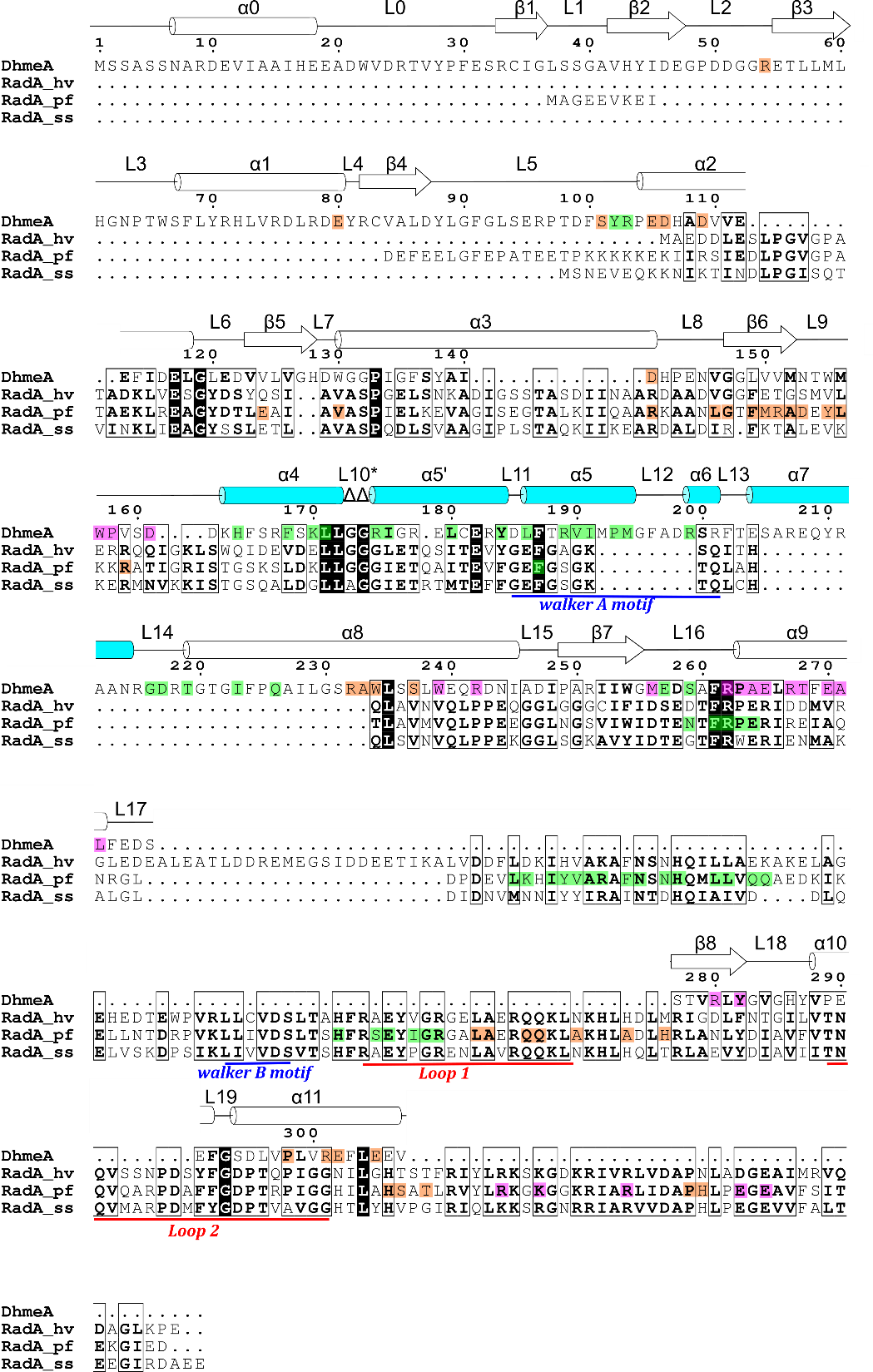


**Fig. S5.** Sequence alignment of DhmeA from *Haloferax mediterranei* with the recombinases: RadA_hv from *Haloferax volcanii*,^13^ RadA_pf from *Pyrococcus furiosus* (RPDB ID: 1pzn^11^), and RadA_ss from *Saccharolobus solfataricus* P2 (PDB ID: 2bke^14^). Identical residues are presented as bold-white letters on a black background, similar residues as bold-black letters on white background. The secondary-structure elements of DhmeA^ΔGG^ are shown above the sequences. The residues forming di-, tetra-, and oligomerization interfaces of are marked with pink, green, and orange background, respectively. Walker A, B and Loop 1, 2 important for DNA binding are marked below the recombinase sequences.

**Supplementary Note 1:**

The dimerization interface of DhmeA^ΔGG^ is symmetrical and it is formed via H-bonds and hydrophobic or non-polar interactions as presented by **Fig. 6A** in the main text. The chain A is coloured in blue and represents the chain with partially unfolded cap domain. The chain B is coloured in yellow and represents the chain with a folded cap domain.

The side chain of R265 (chain A) and the main chain of P158 (chain B) form H-bond with the distance of 2.9 Å. Two atoms of E268 (chain A) from the side chain and the main chain form H-bonds with the side chain of W237 (chain B) and the side chain of R240 (chain B) with the distances of 2.9 and 2.5 Å, respectively. The side chain of D161 (chain A) forms H-bonds with the side chains of Y280 (chain B) and R278 (chain B) with the distances 2.6 and 2.8 Å, respectively. The side chain of T266 from chain A interacts with the same residue from chain B, forming the H-bond with the distance of 3.2 Å. Overall, the dimerization interface is symmetrical; at the same time, residues listed for chain A are the residues for chain B.

The coordination is supported by hydrophobic and non-polar interactions. The following residues, identical for both chains, are involved: W157, R260, A262, and A269. Additionally, four residues from only chain A contribute to hydrophobic interface: M254, P261, E263 and L270.

Overall, approximately 16 amino acids per chain are involved in the formation of the dimerization interface. All ten dimerization interfaces appearing in the ring-like structure of DhmeA show the same set of interactions with similar distances.

**Supplementary Note 2:**

The tetramer of DhmeA^ΔGG^ is formed by two homodimers: AB and CD. The interface between chains A and C, and chains B and D is termed as the tetramerization interface. The interface is formed via H-bonds and hydrophobic or non-polar interactions (**Fig. 6B** of the main text).

Namely, the main chain of V188 (chain C) forms an H-bond with the side chain of Q224 (chain A) with the length of 3.3 Å. The main chain of S257 and the side chain of E255 (chain C) form two H-bonds with the main chain and the side chain of K170 (chain A) with the distances of 2.9 and 2.6 Å, respectively. Side chain of R187 (chain C) forms two H-bonds with the side chain of D215 (chain A) with the lengths of 3.2 and 2.9 Å.

Hydrophobic or non-polar interactions are formed by the following residues of chain C: H164, F168, L171, L172, L178, Y182, L184, I189, P191, M192, and R197. Residues of chain A contributing to the hydrophobic interactions are: Y102, R103, L172, R173, I174, G214, T217, and I221.

The identical set of interface residues listed for chain A are also listed for chain D. The same is true for chains C and B. This interacting pattern with similar distances is found in all five tetramers inside the ring-like structure of DhmeA^ΔGG^.

**Supplementary Note 3:**

The multimerization interface of DhmeA^ΔGG^ is formed between two homo-tetramers (**Fig. 6C** of the main text). Tetramer 1 contains chains A-D and tetramer 2 contains chains E-H. An oligomerization interface between these two tetramers is formed by chains C and E, and chains D and F. The interface is formed via H-bonds or salt bridges, and hydrophobic or non-polar interactions.

Namely, the side chain of R54 (chain D) forms two H-bonds with the main chain of E105 (chain F) and the side chain of D106 (chain F) with the distances 2.9 and 2.8 Å, respectively. In addition, the side chain of R54 (chain D) forms salt bridges with the side chain of D109 (chain F). The side chain of E300 (chain D) forms two H-bonds with the side chain of R230 (chain F) with the distances 2.6 and 2.8 Å. H306 and H307 are the remaining of the cleaved His_6_-tag and contribute to the oligomerization interface: the side chain of H306 (chain D) forms H-bond with the side chain of D109 (chain F) with the length of 2.8 Å. The side chain of H307 (chain D) forms H-bond with the side chain of S235 (chain F) with the distance of 3.2 Å and salt bridges with the side chain of D141 (chain F).

The hydrophobic interactions are formed by S101, R103, A231, and W232 of chain F and E80, P296, R299, and E303 of chain D.

Following the analogy of the tetramerization interface, the diagonally positioned chains contribute the same residues to the formation of the interface. Hence, chains C and F contribute the same set of residues to the oligomerization interface and the same is true for chains E and D. The same interacting pattern is found in all other four multimerization interfaces of the ring-like structure of DhmeA^ΔGG^.

**References:**

1. Sumbalova L, Stourac J, Martinek T, Bednar D, Damborsky J (2018) HotSpot Wizard 3.0: web server for automated design of mutations and smart libraries based on sequence input information. Nucleic Acids Res. 46: W356-W362.

2. Lahoda M, Mesters JR, Stsiapanava A, Chaloupkova R, Kuty M, Damborsky J, Kuta Smatanova I (2014) Crystallographic analysis of 1,2,3-trichloropropane biodegradation by the haloalkane dehalogenase DhaA31. Acta Crystallogr. D Biol. Crystallogr. 70: 209-217.

3. Verschueren KHG, Seljee F, Rozeboom HJ, Kalk KH, Dijkstra BW (1993) Crystallographic analysis of the catalytic mechanism of haloalkane dehalogenase. Nature 363: 693-698.

4. Prokop Z, Sato Y, Brezovsky J, Mozga T, Chaloupkova R, Koudelakova T, Jerabek P, Stepankova V, Natsume R, van Leeuwen JG, Janssen DB, Florian J, Nagata Y, Senda T, Damborsky J (2010) Enantioselectivity of haloalkane dehalogenases and its modulation by surface loop engineering. Angew. Chem. Int. Ed. Engl. 49: 6111-6115.

5. Chaloupkova R, Prudnikova T, Rezacova P, Prokop Z, Koudelakova T, Daniel L, Brezovsky J, Ikeda-Ohtsubo W, Sato Y, Kuty M, Nagata Y, Kuta Smatanova I, Damborsky J (2014) Structural and functional analysis of a novel haloalkane dehalogenase with two halide-binding sites. Acta Crystallogr. D Biol. Crystallogr. 70: 1884-1897.

6. Novak HR, Sayer C, Isupov MN, Gotz D, Spragg AM, Littlechild JA (2014) Biochemical and structural characterisation of a haloalkane dehalogenase from a marine *Rhodobacteraceae*. FEBS Lett. 588: 1616-1622.

7. Chrast L, Tratsiak K, Planas-Iglesias J, Daniel L, Prudnikova T, Brezovsky J, Bednar D, Kuta Smatanova I, Chaloupkova R, Damborsky J (2019) Deciphering the Structural Basis of High Thermostability of Dehalogenase from Psychrophilic Bacterium *Marinobacter* sp. ELB17. Microorganisms 7: 498.

8. Mazur A, Prudnikova T, Grinkevich P, Mesters JR, Mrazova D, Chaloupkova R, Damborsky J, Kuty M, Kolenko P, Kuta Smatanova I (2021) The tetrameric structure of the novel haloalkane dehalogenase DpaA from *Paraglaciecola agarilytica* NO2. Acta. Crystallogr. D Struct. Biol. 77: 347-356.

9. Katoh K, Standley DM (2013) MAFFT multiple sequence alignment software version 7: improvements in performance and usability. Mol. Biol. Evol. 30: 772-780.

10. Robert X, Gouet P (2014) Deciphering key features in protein structures with the new ENDscript server. Nucleic Acids Res. 42: W320-324.

11. Shin DS, Pellegrini L, Daniels DS, Yelent B, Craig L, Bates D, Yu DS, Shivji MK, Hitomi C, Arvai AS, Volkmann N, Tsuruta H, Blundell TL, Venkitaraman AR, Tainer JA (2003) Full-length archaeal Rad51 structure and mutants: mechanism for RAD51 assembly and control by BRCA2. The EMBO Journal 22: 4566-4576.

12. Kinebuchi T, Kagawa W, Enomoto R, Tanaka K, Miyagawa K, Shibata T, Kurumizaka H, Yokoyama S (2004) Structural basis of octameric ring formation and DNA interaction of the human homologous-pairing protein Dmc1. Molecular Cell 14: 363-374.

13. Patoli BB, Winter JA, Patoli AA, Delahay RM, Bunting KA (2017) Co-expression and purification of the RadA recombinase with the RadB paralog from *Haloferax volcanii* yields heteromeric ring-like structures. Microbiology 163: 1802-1811.

14. Ariza A, Richard DJ, White MF, Bond CS (2005) Conformational flexibility revealed by the crystal structure of a crenarchaeal RadA. Nucleic Acids Res 33: 1465-1473.
